# *Pseudoalteromonas* is a symbiont of marine invertebrates that exhibits broad patterns of phylosymbiosis

**DOI:** 10.1093/ismejo/wrag091

**Published:** 2026-04-20

**Authors:** Alejandro De Santiago, Shelby Barnes, Tiago José Pereira, Mirayana Marcellino Barros, Lekeah Durden, Min Khant Han, J Cameron Thrash, Holly M Bik

**Affiliations:** Institute of Bioinformatics, University of Georgia, Athens GA 30605, United States; Department of Marine Sciences, University of Georgia, Athens, GA 30605, United States; Department of Biological Sciences, University of Southern California, Los Angeles, CA 90089, United States; Institute of Bioinformatics, University of Georgia, Athens GA 30605, United States; Department of Marine Sciences, University of Georgia, Athens, GA 30605, United States; Department of Ecology, Evolution, and Organismal Biology, Kennesaw State University, Kennesaw, GA 30144, United States; Department of Marine Sciences, University of Georgia, Athens, GA 30605, United States; Institute of Bioinformatics, University of Georgia, Athens GA 30605, United States; Department of Marine Sciences, University of Georgia, Athens, GA 30605, United States; Department of Marine Sciences, University of Georgia, Athens, GA 30605, United States; Department of Biological Sciences, University of Southern California, Los Angeles, CA 90089, United States; Institute of Bioinformatics, University of Georgia, Athens GA 30605, United States; Department of Marine Sciences, University of Georgia, Athens, GA 30605, United States

**Keywords:** pangenome, *Pseudoalteromonas*, marine invertebrates, microbial evolution, phylosymbiosis

## Abstract

Despite growing insights into the composition of marine invertebrate microbiomes, our understanding of their ecological and evolutionary patterns remains poor, owing to limited sampling depth and low-resolution datasets. Previous studies have provided conflicting results that both confirm and deny the existence of phylosymbiosis between marine invertebrates and marine bacteria. Here, we investigated potential animal–microbe symbioses in *Pseudoalteromonas*, a bacterial genus consistently identified as a core microbiome taxon in diverse invertebrates. Using a pangenomic analysis of 236 free-living and invertebrate-associated bacterial strains (including two new nematode-associated isolates generated in this study), we confirm that *Pseudoalteromonas* is a symbiont with substantial evidence of phylosymbiosis across at least three marine invertebrate phyla (e.g. Nematoda, Mollusca, and Cnidaria). Patterns of symbiosis were consistent irrespective of geography (including in Antarctica), with fluorescence *in situ* hybridization images from nematodes indicating that bacterial symbionts form biofilms in the mouth and esophagus and are sometimes present in female nematode ovaries exhibiting stunted development. The evolutionary history of *Pseudoalteromonas* is marked by substantial host-switching and lifestyle transitions, and host-associated genomes suggest that these bacteria are facultative symbionts involved in nutritional symbioses. In marine environments, we hypothesize that horizontally acquired symbionts may have co-evolved with invertebrates, using host mucus as a physical niche and food source, while providing their animal hosts with vitamin B, amino acids, and bioavailable carbon compounds in return.

## Introduction

The host-associated microbiome has emerged as a key component that underpins the ecology and evolution of diverse eukaryote taxa, from large vertebrates down to microscopic ants [1]. In marine ecosystems, bacterial symbioses have enabled animal hosts such as mollusks, sponges, corals, crustaceans, and annelid worms to colonize extreme deep-sea habitats such as hydrothermal vents and methane seeps, and achieve success in oligotrophic tropical ecosystems, such as coral reefs and seagrass meadows [2, 3]. In the surface ocean, microbial symbioses have been identified as an important driver of biological diversification and speciation in diverse plankton taxa [4], suggesting that the microbiome can facilitate host adaptation to changing environmental conditions and competition over evolutionary time. However, our knowledge of marine symbioses is likely quite incomplete—the majority of studies are restricted to larger taxa in a handful of marine environments, and the broader importance of symbionts is only recently coming to light with large-scale analyses from global efforts such as *Tara* Oceans [4]. In particular, we have yet to explore symbiosis in the majority of marine invertebrate species, and host-microbe evolutionary relationships are largely unknown in smaller-sized meiofaunal animals with a body size <1 mm (e.g. nematodes, tardigrades, kinorhynchs [5, 6]).

Phylosymbiosis is commonly defined as “microbial community relationships that recapitulate the phylogeny of their host” [1], but this strict definition is often expanded to include cophylogenetic signals (where related hosts interact with closely related bacterial taxa, often producing complex cophylogenetic topologies). Here, we use the term “cophylogenetic signal” to refer to broad topological patterns that are shared between host and microbial phylogenies and use this phrase as an interchangeable synonym for “phylosymbiosis.” Cophylogenetic signals can be produced by various evolutionary patterns (as previously defined [7]), including host–symbiont codiversfication (where two or more interacting species undergo reciprocal natural selection), trait-matching (where hosts interact more frequently with bacterial taxa that contain a specific trait of function), or vicariance (when the host and bacteria interact as long as they occupy the same biogeographic area). Compared to the well-defined evolutionary constraints and genome reductions of vertically transmitted symbionts, assessing phylosymbiosis in marine invertebrates is particularly challenging owing to the more “diffusible” nature of marine environments compared to terrestrial habitats (e.g. the faster dilution and transport of bacterial cells and metabolites in water [8–10]), the likely co-occurrence of specialist symbiont taxa alongside more generalist invertebrate microbiome assemblages [8, 9], and the presumed horizontal transmission of many putative marine symbionts [10]. These factors complicate efforts to identify clear genomic signatures of marine symbiosis and distinguish host-associated bacteria from free-living species [11–13]. In addition, marine invertebrate host species can flexibly acquire locally adapted symbiont strains [14, 15] or consistently associate with the same symbiont lineage on a global scale [16], indicating that geographic and evolutionary patterns of symbiosis are highly variable and must be examined at the species level for both the host and microbe.

Does phylosymbiosis exist in marine invertebrate microbiomes? While there have been growing insights into the composition of marine invertebrate microbiomes using 16S ribosomal RNA (rRNA) marker gene surveys [17–19], our understanding of the broader evolutionary and ecological patterns that govern marine microbiome assembly remains poor. The largest study carried out to date found no evidence of cophylogenetic signal across ~1000 marine invertebrate microbiomes spanning 21 animal phyla, despite invertebrate microbiomes clustering separately overall from environmental microbial assemblages [20]. In contrast, more targeted studies have found compelling evidence of phylosymbiosis in diverse animal taxa such as sponges [9, 21], corals [9, 21, 22], nemertean worms [18], and brachyuran crabs [23], even when using data from a single marker gene. These conflicting patterns suggest that 16S rRNA gene surveys are inadequate for evaluating cophylogenetic signal in marine environments, owing to the low resolution of marker gene datasets and an unfavorable signal-to-noise ratio (rRNA datasets also capture signals from extracellular DNA, gut contents, and transient microbiome taxa alongside any putative symbiont taxa [24, 25]). Taken together, the above issues highlight that evaluating phylosymbiosis in marine invertebrates remains a major challenge. There is a critical need for targeted genome-scale studies to investigate phylosymbiosis in marine invertebrates, incorporating expanded sampling of animal hosts as well as phylogenomic analyses of suspected bacterial/archaeal symbiont lineages.

Bacterial groups previously identified as “core microbiome taxa” in 16S rRNA metabarcoding studies represent an ideal starting point for deeper investigations of phylosymbiosis in marine invertebrates [9]. In the present study, we combine new cultured isolates and pangenomic analyses to investigate *Pseudoalteromonas* as a ubiquitous bacterial genus that may represent a common symbiont of marine invertebrates, with high potential for host–microbe coevolution. *Pseudoalteromonas* is consistently recovered as an abundant (and often, statistically significant) taxon in diverse invertebrate microbiomes, including marine nematodes [26, 27], gelatinous zooplankton [19, 28, 29], corals [30, 31], sponges [32], shrimp [33], mollusks [34], and even some protists, including bloom-forming dinoflagellates [35]. *Pseudoalteromonas* bacteria are ubiquitous across marine ecosystems, comprising free-living and particle-associated taxa (isolated from surface ocean waters down to deep-sea sediments; [36, 37]) as well as host-associated species, with the first cultured isolate originally obtained from a marine alga in 1957 [38]. Subsequent host-associated isolates have been acquired from pelagic and benthic marine invertebrates, including both sessile and mobile species [39] as well as bony fish [40]. Similar to other well-characterized symbionts [41], *Pseudoalteromonas* species are characterized by a high degree of bioactivity, producing a variety of secondary metabolites and antibiotics that inhibit the growth of other bacterial taxa [39, 42]. *Pseudoalteromonas* are often found as mucus-associated taxa in corals [8, 9] and have even been observed as the sole bacteria able to colonize marine nematode mucus tracts (suggesting exploitation of mucus as a unique physical niche [27]). *Pseudoalteromonas* is also known to influence the development and fitness of several marine invertebrate species: stimulating immune response, enhancing resistance to pathogenic infections, increasing the digestive enzyme activity of *Apostichopus japonicus* sea cucumber hosts [43], and containing genomic pathways that induce larval settlement and metamorphosis of several marine invertebrate species including the upside-down jellyfish *Cassiopea xamachana* [29], the tubeworm *Hydroides elegans* [44, 45], and the mussel *Mytilus coruscus* [46]. Furthermore, several *Pseudoalteromonas* species are known pathogens of corals and sponges [47–49], suggesting that this host–bacterial relationship is not exclusively beneficial. Over evolutionary timescales, *Pseudoalteromonas* bacteria may have established species-specific relationships with diverse marine invertebrates, deploying robust metabolic toolkits to establish a host-associated niche and providing distinct benefits to (or sometimes negatively exploiting) their marine invertebrate hosts.

In this study, we carry out a pangenomic analysis of 236 free-living and invertebrate-associated *Pseudoalteromonas* bacterial strains (including two nematode-associated isolates generated as part of this study from oncholaimid worms sampled from Tybee Island, GA, USA). We used genome-scale data to broadly assess patterns of phylosymbiosis across diverse marine invertebrate phyla, using two independent statistical metrics to evaluate host–microbe cophylogenetic signals (generalized Robinson–Foulds (RF) and Procrustean approach to test for congruence). Additionally, we utilized metagenome read-mapping approaches to detect *Pseudoalteromonas* signals in single-nematode microbiome datasets spanning three ocean basins (Pacific, Atlantic, and Southern Ocean), coupled with fluorescence *in situ* hybridization (FISH) approaches in live nematodes using genus-specific probes with high bacterial specificity. Our goal was to move beyond common 16S rRNA gene metabarcoding approaches to assess whether genome data and integrative methods can be used to uncover new marine invertebrate symbioses in frequently reported core microbiome taxa.

## Materials and methods

Unless otherwise specified, all bioinformatics tools and computational algorithms were run using default parameters. All scripts utilized during this study have been made available on GitHub: https://github.com/BikLab/Pseudoalteromonas.

### Isolation and sequencing of nematode-associated *Pseudoalteromonas*

To isolate and culture nematode-associated bacteria, marine nematodes from the family Oncholaimidae were isolated from sediment samples collected from Tybee Island, GA, UGA in 2020 [50]. Following methods previously described [51], marine nematodes were isolated using the decantation–flotation method [52], and sediment was washed over 45 μm sieves using artificial seawater (Instant Ocean Spectrum Brands, Inc., USA) prepared with Milli-Q ultrapure water. Nematodes were individually picked under a dissection microscope (Olympus SZX16, Olympus Corporation, Tokyo, Japan) and transferred to sterile artificial seawater on a temporary slide mount. Nematodes were then taxonomically identified under an Olympus BX63 compound microscope to the genus level following the World Register of Marine Species (WoRMS) database [53] using specialized morphological keys for marine nematodes [54–57], placed in a microcentrifuge tube with sterile artificial seawater (Instant Ocean Spectrum Brands, Inc., USA), and immediately shipped live on ice to the University of Southern California, where the sample underwent dilution-to-extinction isolation and were later genome-sequenced, as previously described [50].

### Isolation of marine nematodes for single-worm metagenomics

To identify whether *Pseudoalteromonas* constitutes part of the core nematode microbiome, marine nematodes were isolated from diverse habitats and locations to undergo short-read metagenomic sequencing. Nematodes from Bodega Bay, CA, and the continental shelf of Antarctica were either bulk frozen or stored in DESS [dimethyl sulfoxide (DMSO), ethylenediaminetetraacetic acid (EDTA), and saturated sodium chloride (NaCl) solution], respectively. Nematodes collected from Tybee Island, GA, were morphologically identified and processed when the worms were still alive (in parallel to the culturing experiment described above). All nematodes were isolated from sediments using a decantation–flotation method [52]. Samples were washed over a 45 μm sieve using artificial seawater (Instant Ocean Spectrum Brands, Inc., USA) prepared with Milli-Q ultrapure water. Nematodes were individually picked under a dissection microscope and transferred to sterile artificial seawater on a temporary slide mount. Individual worms were then taxonomically identified to the family or genus level following the WoRMS database [53] using specialized morphological keys for marine nematodes [54–57] as described above. Nematodes were quickly rinsed in molecular-grade water following methods previously described to remove transient environmental microbes [58] before being stored in microcentrifuge tubes with either 20 μl of water or 10 μl REPLI-g Single Cell Cryo-protect Reagent (QIAGEN). One nematode specimen was placed in each tube to characterize the microbiome associated with each worm. Lab workspaces and equipment were sterilized with 70% ethanol before and between samples. Nematodes from Antarctica were frozen in DESS [59] and transferred to 10 μl REPLI-g Single Cell Cryo-protect Reagent (QIAGEN) prior to DNA extraction and amplification.

Worms initially placed in 20 μl of molecular-grade water underwent DNA extraction using the E.Z.N.A MicroElute Genomic DNA Kit following the manufacturer’s protocol. Worms in 10 μl REPLI-g Single Cell Cryo-protect Reagent (QIAGEN) underwent DNA lysis and multiple displacement amplification (MDA) using the REPLI-g Advanced DNA Single Cell Kit (QIAGEN) with a modified protocol to accommodate storage of the worms (which were stored in 10 μl of REPLI-g Single Cell Cryo-protect Reagent compared to the manufacturer’s recommended 4 μl) and to target the lysis of bacterial cells. A total of 6 μl of Buffer D2, containing 0.5 μl of 1 M dithiothreitol (DTT) and 5.5 μl of reconstituted direct lysis buffer (DLB), was added to the nematodes stored in 10 μl REPLI-g Single Cell Cryo-Protect Reagent (QIAGEN) to lyse the nematode-associated bacterial cells. Tubes were vortexed and centrifuged to briefly mix the samples. The reaction mixture was incubated in a Thermomixer heated shaker block (Eppendorf, Hamburg, Germany) at 65°C for 10 min. A total of 6 μl of Stop Solution was added to each tube. Amplification reactions were 50 μl total reaction, containing 9 μl of water, 29 μl of REPLI-g advanced sc Reaction Buffer, 2 μl of REPLI-g DNA polymerase, and 10 μl of denatured DNA. The samples were incubated in a T1000 Thermocycler (Bio-Rad) at 30°C for 2 h with a lid temperature of 70°C, followed by 65°C for 2 min to inactivate the amplification reaction. Samples that underwent successful amplification or had high DNA biomass during the initial lysis step were sent for library preparation and sequencing using the NovaSeq 6000 System (Illumina) at the Department of Energy Joint Genome Institute or the NextSeq 2000 System (Illumina) at the Georgia Genomics and Bioinformatics Core at the University of Georgia. A subset of nematode genera, representing four distinct families, was included in this study (Table S2).

### Assembling the *Pseudoalteromonas* pangenome

A total of 236 *Pseudoalteromonas* genomes, including all 234 high-quality genomes available in RefSeq (completeness >95%; contamination <5%) as of August 2024 and two nematode-associated genomes generated as part of this study, were used to assemble the *Pseudoalteromonas* pangenome (Table S1). The collection consisted of 91 genomes from bacteria isolated from seawater (“free-living” isolates) and 145 genomes isolated from marine invertebrates (“host-associated” isolates). We performed gene-calling and annotation using PROKKA v1.14.5 [60] with UniProt [61] as a protein reference database. PIRATE v1.0.5 [62] was used to identify orthologous genes and assemble the pangenome using the default percent identity thresholds recommended by the developers. A custom RScript (available on GitHub) was used to group orthologous gene families into core (genes present in >95% of the genomes), accessory (genes present in <95% and found in more than one genome), and unique genes (genes present in only one genome). Pearson’s chi-square statistical test was used to test if there was a relationship between the frequency of genes (core, accessory, and unique) and lifestyle. To determine whether *Pseudoalteromonas* exhibited an open or closed pangenome, we calculated gene accumulation curves of the gene families using a custom RScript (also available on GitHub at the repository linked above). The pangenome was designated as “open” if new gene families were identified as new genomes were added (i.e. the accumulation curve did not reach an asymptote). The pangenome was classified as “closed” if no new gene families were found (i.e. the accumulation curve reached an asymptote).

### Inferring the *Pseudoalteromonas* phylogenomic tree and population structure

To infer a pangenome-based phylogeny of *Pseudoalteromonas*, PIRATE v1.0.5 [62] was used to identify and individually align the 1689 core genes using MAFFT v7.5.20 [63] and subsequently concatenate them into a nucleotide core alignment. The core alignment was used to infer a maximum-likelihood phylogenetic tree with RAxML v8.2.12 [64] using the General Time-Reversible (GTR) model with GAMMA (+Γ)distributed rates across sites and 10 000 bootstraps. The dRep v3.4.2 [65], with the fastANI algorithm [66], was used to compare genomic relatedness and cluster genomes with 95% similarity into phylogenetic groups.

To identify whether the accessory genes were shared between phylogroups or lifestyles, a genome–genome network was constructed. The PIRATE presence/absence matrix was processed using the GraPPLE toolkit [67] to explore the accessory pangenome. The presence/absence matrix was filtered to include only the accessory genes present in >5% of the bacterial genomes. Core and unique gene families were excluded from this analysis. The presence/absence matrix was converted into a binary format using the GraPPLE script “gene_matrix_to_binary.py.” The GraPPLE script “pw_similarity.py” was run to calculate pairwise similarities between genomes to create a genome-genome network and compare genomic similarities between lifestyle (host-associated vs. free-living) and phylogroups. Finally, the genome-genome network graphs were visualized using the Graphia v4.2 platform [68].

### Evaluating phylosymbiosis among *Pseudoalteromonas* spp. and marine invertebrate hosts

Following the methods used in previous studies of bacterial–animal phylosymbiosis [69], midrooted phylogenetic trees were inferred using the core alignments for Mollusca-, Cnidaria-, and Porifera-associated *Pseudoalteromonas*. This analysis was restricted to these phyla due to insufficient genomic representatives across other invertebrate phyla. The alignments were used to infer a maximum-likelihood phylogenetic tree with RAxML v8.2.12 [64] using the GTR model with GAMMA-distributed rates across sites and 10 000 bootstraps. The host phylogenies (i.e. Mollusca, Cnidaria, and Porifera) were inferred using the TimeTree Database v5 [70].

A cophylogenetic signal was defined as a nonrandom association between the host and symbiont phylogenies [7, 71]. One specific scenario, phylogenetic congruence, was assessed as a distinct example of a cophylogenetic signal characterized by shared patterns of codiversification between the host and its symbiont [7, 71]. The cophylogenetic signal was tested using two methods: (i) PACo (a Procrustean Approach to test for congruence of two phylogenetic trees [72]) using the paco R package and (ii) using the Generalized RF metric [73] implemented in the TreeDist R package [74]. The PACo analysis uses a patristic (tip-to-tip branch lengths) distance matrix, which is not impacted by rooting methods [75], to compare two phylogenetic trees. In contrast, the generalized RF metric compares the number of splits that co-occur between two unrooted phylogenetic trees [74]. The cophylogenetic signal was determined if the PACo analysis, with 1000 random permutations between the host and symbiont phylogenetic trees, was significant (*P*-value <.05). Evidence of cophylogenetic signal was determined using the generalized RF metric and PACo Coefficient of Determination (*R*^2^). *R*^2^ was calculated from the Procrustes squared sums (*R*^2^ = 1 − m^2^). A high *R*^2^ (1) indicates perfect phylogenetic congruence. Following the recommendations of Perez-Lamarque and Morlon [71], phylogenetic congruence was determined if the PACo analysis was significant with an *R*^2^ > 0.25 and the generalized RF metric was used to identify if evidence of the phylogenetic congruence was weak (generalized RF = 0.25–0.75) or strong (generalized RF < 0.25).

To further visualize phylogenetic and geographic patterns seen in nematode-associated *Pseudoalteromonas* strains, we used a read-mapping approach whereby single-worm metagenomes (e.g. low-complexity metagenomes containing only a single host genome and its associated microbiome community) were mapped to the *Pseudoalteromonas* pangenome. The quality of the sequences was visualized using FastQC [76] and summarized using MultiQC [77]. Sequences were quality trimmed and underwent adaptor removal using Trimmomatic [78] with a sliding window (4:15) and a minimum length of 36 bp. Kraken2 [79], was used to identify and extract bacterial reads from the nematode/microbiome metagenomic dataset. The quality-controlled sequences were mapped to *Pseudoalteromonas* genomes using the Read Recruitment Analysis Pipeline (RRAP) [80]. RRAP normalizes the number of mapped reads by sequencing depth and genome size by using the RPKM (reads per kilobase [of the genome] per million [bases of recruited sequences]) method. Samples with total RPKM <1 were removed, and a minimum RPKM threshold of 0.1 was implemented to account for false positives. The results were visualized as a heatmap and phylogenetic framework using the ggtree [81] package in R. The PACo analysis was used to test for cophylogenetic signals between the nematode phylogeny and the hierarchical clustering of the read mapping dissimilarity matrix using the Bray–Curtis distance matrix.

### Identifying metabolic pathways and secondary metabolites across the pangenome

METABOLIC-G v4.0 [82] was run with the full KOfam database [83] to predict the metabolic and biogeochemical traits of each bacterial genome included in this study. A multiSMASH (https://github.com/zreitz/multismash) snakemake pipeline was used to summarize the secondary metabolite biosynthesis gene clusters predicted by the antiSMASH software [84]. The presence/absence of the METABOLIC and antiSMASH results were imported into R for downstream data analysis. METABOLIC and antiSMASH results were visualized in a phylogenetic framework using the ggtree package [81] in R. The Scoary2 software [85] was used to conduct a pangenomic genome-wide association study (GWAS) to identify genes (using the pangenomic presence/absence table from PIRATE) and functions (using the METABOLIC and antiSMASH results) that co-occur with the bacterial lifestyles (i.e. host-associated or free-living) and phylogroups. Scoary2 determines significant trait-associated genes using a Fisher’s test (*P*-value <.05) with a Bonferroni adjustment using a family-wise error rate (FWER) cutoff of 0.99 to reduce the false-positive discovery rate. Hypothetical proteins predicted by Prokka were annotated by Gaia, an artificial intelligence (AI) tool developed by Tatta Bio, that integrates several genomic and structural features to predict functions of hypothetical proteins (https://gaia.tatta.bio/) [86].

### Visualizing *Pseudoalteromonas* bacteria in nematodes using fluorescence *in situ* hybridization

*Oncholaimidae* nematodes collected from the Florida Keys and Tybee Island, GA, were processed using a modified protocol previously used for FISH of nematode-associated bacteria [26]. Nematodes belonging to the family Oncholaimidae were fixed for 2 h in 3% formaldehyde with sterile artificial seawater (Instant Ocean Spectrum Brands, Inc., USA), followed by three rinses in a 1:1 solution of Phosphate-Buffered Saline (PBS):sterile seawater. The samples were stored in 1:1 storage buffer (100% ethanol:2× PBS) and kept frozen at −20°C until they were used. Nematodes were rinsed in 30% formamide buffer and hybridized in 150 μl hybridization buffer [0.9 M NaCl, 0.02 M Tris-HCl (pH 8.0), 0.01% SDS, 2 ml blocking reagent [5% bovine serum albumin (BSA in 0.5% PBS-Tween 0.5%) and 95% 7.4 pH PBS], and 30% formamide] at 46°C for 3 h with 15 μl (8 μM) of each probe: a combination of universal bacterial probes that has been shown to increase coverage of bacterial phyla (EUB338I: 5′-GCTGCCTCCCGTAGGAGT-3′; CY-3; [87]; EUB338II: 5′-GCAGCCACCCGTAGGTGT-3′; CY-3; [87, 88]), *Pseudoalteromonas*-specific probe (5′-TTGACCCAGGTGGCTGCC-3′; CY-5; [89]), and nonsense probe (5′-ACTCCTACGGGAGGCAGC-3′; FAM; [90]). The *Pseudoalteromonas* probes were tested *in silico* using the SILVA TestProbe Software and were found to have a 100% specificity and 87.5% coverage to the *Pseudoalteromonas* sequences in the SILVA v132 16S rRNA database. The nematodes were rinsed three times in washing buffer [0.046 M NaCl, 0.02 M Tris-HCl (pH 80), 0.005 M Ethylenediaminetetraacetic acid (EDTA), 0.01% SDS] for 30 min at 46°C for a total of 1.5 h. The hybridized specimens were mounted on a slide in SlowFade Gold Antifade reagent (Invitrogen) containing 4′,6-diamidino-2-phenylindole (DAPI) and observed using the Zeiss LSM 880 Confocal Microscope system. The Zen 2.3 imaging software was used for image acquisition and processing. Images were acquired as single-plane confocal images using a 40× oil-immersion objective. The whole-nematode image was generated as tiled acquisitions using identical imaging parameters. All images were subsequently processed using FIJI (ImageJ).

## Results

### Expanded pangenomic view of the ubiquitous bacterial genus *Pseudoalteromonas*

**Figure 1:**
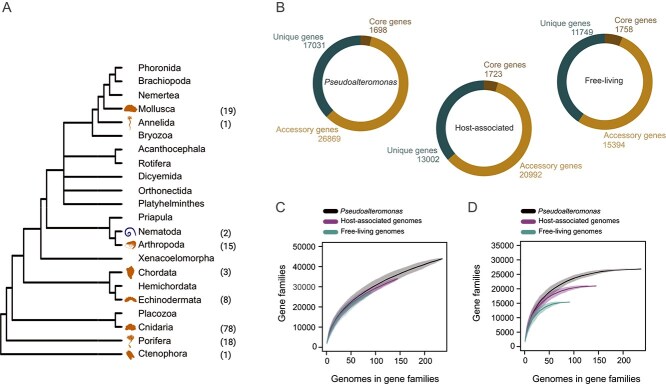
*Pseudoalteromonas* has an open pangenome that is driven by the accumulation of unique genes. (A) The phylogenetic tree of marine invertebrates was adapted from a previous study [20]. The Blue PhyloPics (e.g. Nematoda) indicate host-associated *Pseudoalteromonas* that were isolated from this study, and gold PhyloPics (e.g. Annelida, Arthropoda, Chordata, Cnidaria, Ctenophora, Echinodermata, Mollusca, and Porifera) indicate the publicly available host-associated *Pseudoalteromonas* genomes included in this study. Parenthetical numbers represent the number strains isolated from each phylum. (B) The number of core genes (brown; 95% of genomes), accessory genes (gold; present <95% of genomes), and unique genes (green) found in all *Pseudoalteromonas*, host-associated, and free-living pangenomes. (C) Accumulation curves of the three pangenomes indicate that *Pseudoalteromonas* has a large pangenome; however, (D) accumulation curves of the accessory genes reach an asymptote, indicating that the pangenome openness is being driven by unique genes.

To characterize the genomic repertoire and functional diversity of the genus *Pseudoalteromonas*, we constructed a pangenome using 234 publicly available genomes and two nematode-associated *Pseudoalteromonas undina* strains isolated as part of this study (Table S1), the latter of which we obtained by adapting high-throughput culturing approaches originally developed for studies of pelagic marine bacteria [50, 91]. *Pseudoalteromonas* has been the target of several comparative pangenomic studies [42, 92, 93]; however, to the best of our knowledge, this is the largest pangenomic study *Pseudoalteromonas* that aims to identify whether cophylogeny exists between *Pseudoalteromonas* and marine invertebrates. Our final pangenome consisted of 91 free-living and 145 host-associated *Pseudoalteromonas* isolated from various marine environments. The host-associated strains were previously isolated from nine marine invertebrate Phyla (Arthropoda, Annelida, Chordata, Cnidaria, Ctenophora, Echinodermata, Nematoda, Mollusca, and Porifera; Fig. 1A). The most common hosts were Cnidaria (78 isolates), followed by Mollusca (19 isolates), and Porifera (18 isolates). The phylum Annelida (1 isolate), Nematoda (2 isolates, both obtained in this study), and Chordata (3 isolates) were the least common animal hosts in our dataset.

The size and distribution of the *Pseudoalteromonas* pangenome was calculated using three parallel approaches: 1) the entire genus, 2) free-living genomes only, and 3) host-associated genomes only. Following a standard approach used to characterize bacterial pangenomes [94], gene families were divided into three groups according to the frequency at which they appear in the pangenome (Fig. 1B): core genes (present in >95% of the genomes), accessory genes (present in <95% of the genomes but not unique to a single genome), or unique genes (found only in one genome). Our results showed that the *Pseudoalteromonas* pangenome could be classified as an open pangenome, characterized by a small set of core genes (3.71%; 1689 genes) and a large number of accessory (59.01%; 26 853) and unique genes (37.28%; 16 962). When partitioned by lifestyle (Fig. 1B), the host-associated pangenome contained 5042 more accessory genes (20970) compared to the free-living pangenome (15 928; *P*-value <2.2e-16). Accumulation curves of the entire pangenome, which include the core, accessory, and unique gene families, did not asymptote, indicating that *Pseudoalteromonas* has an open pangenome and that our expanded pangenomic analysis does not yet capture the true biological diversity of this genus (Fig. 1C). However, accumulation curves of the accessory gene families for the free-living, host-associated, and genus-level pangenomes reached an asymptote (Fig. 1D), indicating that the openness of the pangenome was being driven mainly through the accumulation of unique genes. Our expanded pangenome analysis is consistent with previous findings that identified a high genomic heterogeneity within this bacterial genus, even for closely related *Pseudoalteromonas* strains [42]. These results indicate that *Pseudoalteromonas* lineages maintain a high degree of genomic flexibility, which may facilitate rapid exploitation of diverse ecological niches in the marine environment.

### Pseudoalteromonas exhibits distinct phylogroups and frequent lifestyle transitions

**Figure 2:**
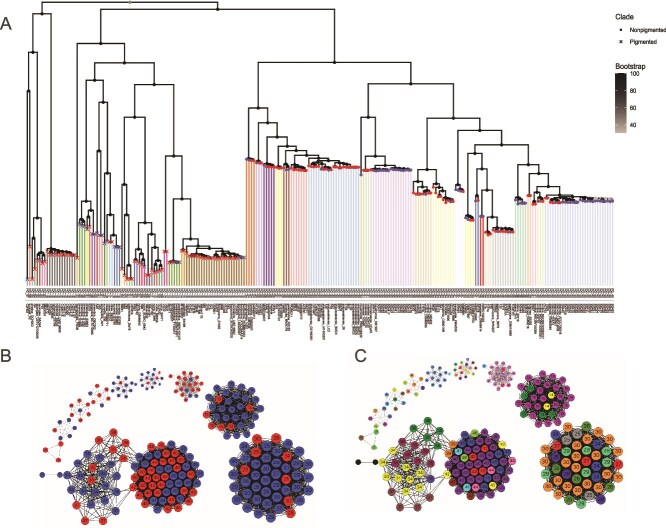
The accessory pangenome of *Pseudoalteromonas* clusters according to phylogroups, rather than bacterial lifestyle. (A) A midpoint-rooted maximum-likelihood phylogenetic tree constructed using the 1689 core genes and 10 000 bootstraps. Tip color indicates whether the bacterial isolate is free-living (blue) or host-associated (red). The shapes indicate if the genome is pigmented (x) or nonpigmented (circles), according to a previous pangenomic study [42]. The color of the lines differentiates the different phylogroups. (B) Genome−genome network analysis using most of the accessory genome (removing accessory genes present in <5% of the genomes). The nodes represent individual genomes, and edges connect genomes that contain similar accessory genes. Red and blue circles indicate host-associated and free-living genomes, respectively. (C) The genome–genome network analysis with samples labeled according to phylogroup. The colors represent phylogroups but are independent of (A).

To unravel the evolutionary dynamics and population structure of free-living and host-associated *Pseudoalteromonas*, core genes (1689) were used to construct a midrooted maximum likelihood phylogenetic tree and genomes were delineated into phylogroups using average nucleotide identity (ANI). We identified 57 distinct phylogroups, substantially increasing the known diversity of *Pseudoalteromonas* beyond the 34 species currently described in the literature (Fig. 2A and Fig. S1). Across the phylogenetic tree, phylogroup membership and clade branching patterns indicated frequent transitions between free-living and host-associated lifestyles as well as substantial host-switching between invertebrate taxa (Fig. 2A), indicative of a flexible genomic repertoire in *Pseudoalteromonas* that facilitates association with diverse hosts and habitats. These evolutionary patterns suggest a “nomadic” lifestyle seen in other bacterial groups (where bacteria maintain a “universal” set of genes that allow them to thrive in a variety of environments [95]), and phylogenetic patterns indicate an absence of clade-specific environmental adaptation. Of the 57 phylogroups, 34.48% (20 phylogroups) comprised a mixture of host-associated and free-living isolates. The majority of the phylogroups (65.52%) were lifestyle-specific, containing only free-living or only host-associated taxa. Of the lifestyle-specific phylogroups, 25 comprised only host-associated genomes, versus fewer than 12 phylogroups with entirely free-living strains. However, the majority of these lifestyle-specific phylogroups (31) contained less than three genomic representatives, suggesting poor taxon sampling and the need for additional sampling efforts to confirm the veracity of these patterns. Overall, the *Pseudoalteromonas* phylogeny was divided into three major clades: a group of closely related bacteria with shorter branch lengths and two smaller clades with longer branches and greater evolutionary divergence (Fig. 2A). The structure and topologies of these three major clades reflect previously described phylogenetic patterns for pigmented and non-pigmented clades of *Pseudoalteromonas* species [42].

**Figure 3:**
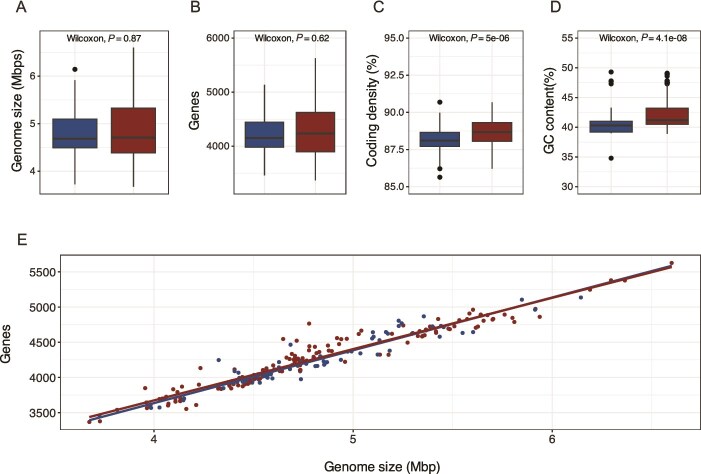
Host-associated lineages exhibit significantly higher coding density and GC content compared to free-living strains. Differences between four different genomic characteristics, including (A) genome size (bp), (B) total number of genes, (C) coding density, and (D) GC content (%) between host-associated and free-living *Pseudoalteromonas* genomes. Differences in coding density and GC content were significant based on Wilcoxon signed-rank tests.

To assess whether the distribution of accessory gene families corresponded to lifestyle (host-associated vs. free-living) or evolutionary history (phylogroups), we conducted a genome-genome network analysis using accessory genes found in at least 5% of the genomes (Fig. 2B and C). The isolates were not grouped by lifestyle but were instead structured by closely related phylogroups (Fig. 2B and C). The genome size (Wilcoxon *P*-value = .87) and number of genes (Wilcoxon *P*-value = .67) between host-associated and free-living genomes were not significantly different (Fig. 3A and B). However, the host-associated genomes exhibited significantly higher coding density (Wilcoxon *P*-value <.01) and a nucleotide bias (higher GC content; Wilcoxon *P*-value <.01) compared to the free-living genomes (Fig. 3C and D). Such evolutionary “jumps” in GC content—including both increases and decreases in GC content—have been increasingly documented in bacterial lineages with host-associated lifestyles [96], although the mechanisms explaining such GC jumps are not well understood.

### Evidence of phylosymbiosis across diverse marine invertebrates

To test for phylosymbiosis between marine invertebrates and *Pseudoalteromonas,* we constructed seven host and bacterial phylogenetic trees: one tree spanning all hosts in the Kingdom Animalia (to evaluate broad patterns of phylosymbiosis, analogous to the approach implemented in previous studies [20]), four phylum-specific trees for invertebrate groups representing the most well-sampled animal phyla in our dataset (Mollusca, Cnidaria, Porifera, and Nematoda), and trees for Class Bivalvia (Mollusca) and Family Faviidae (Cnidaria) representing the two host phylogenies with the most extensive taxon sampling at lower taxonomic levels.

**Figure 4:**
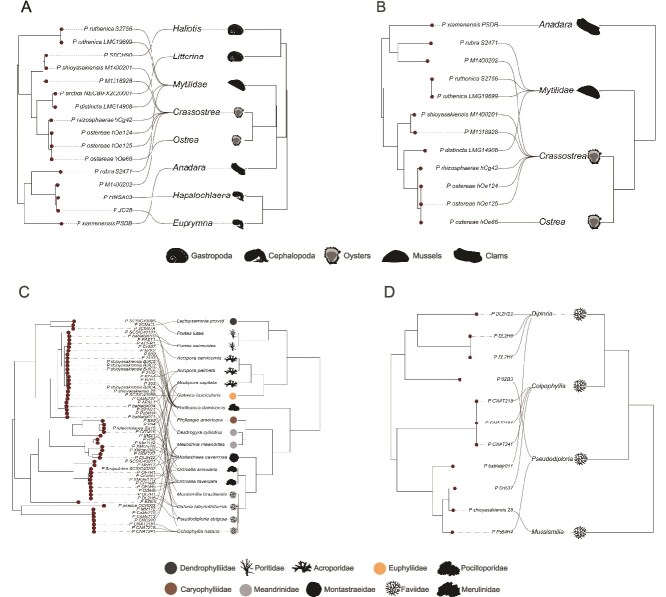
Evidence of cophylogeny and phylogenetic congruence between *Pseudoalteromonas* and marine invertebrate taxa. Phylogenetic trees used to test for cophylogeny among (A) Mollusca, (B) Bivalvia (Mollusca), (C) Cnidaria, and (D) Faviidae (Cnidaria). Cophylogeny signal was tested using PACo, and phylogenetic congruence was tested using the generalized Robinson–Foulds metric (Table 1).

**Figure 5:**
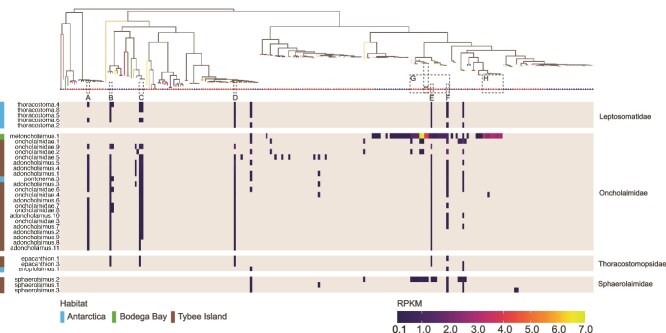
Read-mapping approaches recover consistent signals from *Pseudoalteromonas* symbionts irrespective of geographic region. Heatmap depicts metagenome reads from single-worm sequencing that map to *Pseudoalteromonas* genomes. The *Pseudoalteromonas* bacterial phylogeny is displayed horizontally across the top, with branch coloring indicating phylogroup membership and circles indicating lineages with host-associated (red) or free-living (blue) lifestyles. Black stars indicate the two isolates obtained as part of this study. Nematode metagenomes are labeled on the left-hand side, arranged by taxonomic hierarchy (text on the right indicates family). Marine nematode samples are identified down to family or genus of each individually sequenced worm, and the color indicates the geographic location where each specimen was obtained: Antarctica (blue), Bodega Bay, CA (green), Tybee Island, GA (brown). Gray cells indicate samples that did not read map to the corresponding *Pseudoalteromonas* genome (RPKM < 0.1).

Using genome-scale data, we found consistent evidence of cophylogenetic signals but not phylogenetic congruence at most of the taxonomic levels examined (Fig. 4, Fig. S2, and Table 1), despite *Pseudoalteromonas* genomes being collected across different temporal and spatial scales. At the level of Kingdom Animalia, we found evidence of low cophylogenetic signal (PACo *P*-value <.001; PACo *R*^2^ = 0.07), but not of phylogenetic congruence (generalized RF = 0.78; Fig. S2). This low (but statistically significant) cophylogenetic signal across all animal phyla is likely due to substantial host switching by *Pseudoalteromonas* lineages among phylogenetic groups as phylogenetic congruences and cophylogenetic signals reflect two different evolutionary processes (Fig. S2; [7]). Phylogenetic congruence measures whether the host’s evolutionary history is dependent on the symbiont (or vice versa); however, cophylogenetic signals can emerge if the host (or symbiont) evolutionary history is shaped by conserved traits or biogeography [71]. Statistical support for phylosymbiosis becomes stronger when animal hosts are evaluated at the phylum, class, and family levels. We found evidence of cophylogeny (PACo *P*-value = .012; PACo *R*^2^ = 0.60) and weak phylogenetic congruence (generalized RF = 0.60) between *Pseudoalteromonas* and Mollusca (Fig. 3A and Table 1). In Mollusca, two bacterial lineages belonging to phylogroup PG15 interacted with two cephalopod hosts, whereas PG30 showed evidence of host switching from Crassostrea (true oysters) to Haliotis (abalone sea snails). Within Class Bivalvia (Mollusca), we recovered high cophylogenetic signal (PACo *P*-value = .025; PACo *R*^2^ = 0.41) and weak phylogenetic congruence (generalized RF = 0.75) with *Pseudoalteromonas* (Fig. 3B and Table 1), where five *Pseudoalteromonas* lineages (spanning three phylogroups) interacted with Crassostrea and exhibited host switching with two other bivalve groups, Mytilidae (mussels) and *Ostrea* spp*.* (edible oysters). There was low cophylogenetic signal (PACo *P*-value = .011; PACo *R*^2^ = 0.11) but no phylogenetic congruence (generalized RF = 0.85) between *Pseudoalteromonas* and the phylum Cnidaria (Fig. 3C and Table 1), but high cophylogenetic signal (PACo *P*-value = .025; PACo *R*^2^ = 0.41) and weak phylogenetic congruence (generalized RF = 0.73) for stony corals within Family Faviidae (Fig. 3D and Table 1). Within the Faviidae, three *Pseudoalteromonas* lineages (PG15, PG9, and PG50) interacted closely with *Diploria* spp*.* (grooved brain corals), and five *Pseudoalteromonas* lineages (PG16, PG24, PG26, PG29, PG30) interacted with *Colpophyllia* spp*.* (boulder brain corals). Additionally, the two *Pseudoalteromonas* lineages associated with *Pseudodiploria* spp*.* stony corals demonstrated host switching. Similar to other invertebrate phyla, we saw evidence of high cophylogenetic signal (PACo *P*-value <.001; PACo *R*^2^ = 0.20) between *Pseudoalteromonas* and the phylum Nematoda (Fig. 5 and Table 1) when assessing host/bacterial phylogenetic trees. Only one analysis showed no evidence of cophylogenetic signal or phylogenetic congruence: that between *Pseudoalteromonas* and the phylum Porifera (generalized RF = 0.94; PACo *P*-value = .470; PACo *R*^2^ = 0.14; Table 1). Evidence of phylosymbiosis became stronger (higher cophylogenetic signal and the emergence of statistically significant phylogenetic congruence) when lower-level taxonomic groups were assessed (Class Bivalvia and Family Faviidae). However, analysis at lower taxonomic levels only included four-member host trees, indicating that higher taxon sampling is needed to confirm phylosymbiosis (although four-member trees have previously been sufficient to assess clade-specific phylosymbiosis [97]). A high cophylogenetic signal was also obtained at the phylum level (in Mollusca and Nematoda) when taxon sampling was deep enough for statistical support to emerge. These data suggest that bacterial genome data, in addition to extensive host taxon sampling, are imperative for detecting signals of phylosymbiosis in marine invertebrates. The frequent lifestyle transitions of *Pseudoalteromonas* lineages and apparent host switching seen across marine invertebrates can further obscure signals of phylosymbiosis at the kingdom/phylum level, particularly in previous studies that utilize only low-resolution 16S rRNA data [20].

**Table 1:** Statistical support for phylosymbiosis in marine invertebrates, indicated by cophylogenetic signals and phylogenetic congruence of *Pseudoalteromonas*. Results from the PACo analysis and the generalized Robinson–Foulds (RF) metric indicate whether there is a cophylogenetic signal (PACo *P*-value <.05) and evidence of phylogenetic congruence across marine invertebrate phyla. A generalized RF of 0 indicates perfect congruence between host-associated and bacterial phylogenies.

Host	PACo *P*-value	PACo m^2^	PACo *R*^2^ (1 − m^2^)	Generalized Robinson–Foulds	Cophylogenetic signal	Phylogenetic congruence
Animalia	0.00	0.93	0.07	0.85	Low	None
Mollusca	0.01	0.60	0.40	0.60	High	Weak
Cnidaria	0.01	0.89	0.11	0.85	Low	None
Porifera	0.47	0.86	0.14	0.93	None	None
Nematoda	0.00	0.80	0.20	NA	High	None
Bivalvia (Mollusca)	0.01	0.65	0.34	0.75	High	Weak
Faviidae (Cnidaria)	0.03	0.59	0.41	0.73	High	Weak

To further assess phylosymbiosis between *Pseudoalteromonas* and representatives from the phylum Nematoda, we obtained nematode-associated microbiomes via single-worm sequencing from eight marine nematode genera (representing two orders and four families). Samples were collected from four distinct environments (the Antarctic continental shelf, intertidal sites on the Georgia coast, subtidal coral sands in the Florida Keys, and seagrass habitats in Bodega Bay, CQ Table S2), and bacterial abundances were quantified as Reads Per Kilobase per Million mapped reads (RPKM). Single-nematode read-mapping indicated that the same *Pseudoalteromonas* strains were consistently associated with nematode hosts irrespective of geography: Antarctic nematode microbiomes contained the same *Pseudoalteromonas* lineages as nematode specimens obtained from the Georgia, California, and Florida coastlines (Fig. 5 Clades A–F). We often detected five or more distinct *Pseudoalteromonas* lineages associated with individual marine nematodes (spanning both pigmented and non-pigmented bacterial clades; Fig. 5 Clades A–E), suggesting the existence of complex symbioses involving multiple congeneric bacterial species. *Pseudoalteromonas* read-mapping signals were strongest within the nematode Family Oncholaimidae (Mean RPKM per sample: 6.44) and Sphaerolaimidae (Mean RPKM per sample: 3.44) consistent but weaker in Family Leptosomatidae (Mean RPKM per sample: 2.09), and sporadic or absent in Thoracostomopsidae (Mean RPKM per sample: 1.18); Fig. 5 Clades B–F), further suggesting that associations with *Pseudoalteromonas* may impact the ecology and life history of a specific subset of marine nematode taxa, and these bacteria may have co-evolved with specific evolutionary lineages of nematodes (e.g. Family Oncholaimidae). Of the *Pseudoalteromonas* lineages detected with read-mapping in oncholaimid nematode specimens, three were previously characterized as free-living *Pseudoalteromonas* strains, whereas four were previously isolated from invertebrate hosts (three from Arthropoda and one from Cnidaria; Fig. 5 Clades A–F), indicating that our overall knowledge of *Pseudoalteromonas* habitats and host associations is incomplete. In addition, metagenomic reads from one oncholaimid nematode genus (*Metoncholaimus* sp*.*) mapped to all the strains in several *Pseudoalteromonas* phylogroups (PG45, PG43, PG34, PG35, and PG36), which include free-living and host-associated strains previously isolated from various invertebrate taxa (Arthropoda, Chordata, Cnidaria, Echinodermata, Mollusca, and Porifera), including the two new nematode-associated *Pseudoalteromonas* isolates obtained in this study (Fig. 5 Clades G and H). A single sample from the Family Sphaerolaimidae (Sphaerolaimus.2) mapped to some of the same phylogroups recovered in the *Metoncholaimus* sp*.* nematode (PG43, PG35, PG36), including the two nematode-associated strains obtained in the present study (Fig. 5). These patterns suggest that marine nematodes may associate with a phylogenetically diverse set of *Pseudoalteromonas* lineages spanning many phylogroups (as seen for most marine nematodes in Family Oncholaimidae and Leptosomatidae) or alternatively harbor many closely related *Pseudoalteromonas* lineages restricted to a small number of bacterial phylogroups (as seen in the oncholaimid genus *Metoncholaimus*). Patterns of nematode-associated *Pseudoalteromonas* were consistent within family-level clades and maintained across vast geographic distances, suggesting that these symbionts may have ancient evolutionary origins.

### Functional pathways suggest nutritional symbioses in host-associated lineages

We used a panGWAS [85] approach to identify genes and functions that were more common in host-associated genomes. Due to substantial differences in evolutionary rates between the two major *Pseudoalteromonas* clades (pigmented and nonpigmented clades), we analyzed these major clades separately to identify genes that may underpin host-associated lifestyles, as recommended by a prior pangenomic study [42]. Lifestyle transitions toward symbiosis appear to emphasize genes and pathways related to the breakdown of organic compounds (peptides, cellulose, chitin, and other carbohydrates), production and transport of antimicrobial and antitoxin compounds, biofilm formation, production of essential vitamins, and enhanced transport of vitamin B12.

Of the 26 869 pigmented accessory genes, only 6 were more abundant in the host-associated pigmented strains (three of which were hypothetical proteins; Fig. S3 and Table S3). The three annotated genes included an inner membrane transport protein YhdP (panGWAS odds ratio = 8.75; *P-*value <.001) that maintains outer membrane permeability, an acetoin utilization protein (panGWAS odds ratio = 22.15; *P*-value <.0001), and an helix-turn-helix (HTH)-type transcriptional regulator *DmlR* (panGWAS odds ratio = 8.09; *P*-value = .001) that regulates aerobic growth on *D*-malate (Table S3). The three AI-annotated hypothetical proteins (see Materials and Methods) were predicted to be a periplasmic substrate-binding protein (panGWAS odds ratio = 12.00; *P*-value = .0001), a complex carbohydrate transporter (panGWAS odds ratio = 8.70; *P*-value = .0001), and a gene involved in bacteriocin (antimicrobial peptide) production or immunity (panGWAS odds ratio = 8.413; *P*-value = .0001).

In the nonpigmented genomes, 111 genes were more abundant in the host-associated strains, with 70 classified as hypothetical proteins (Table S3). The annotated genes included three additional HTH-type transcriptional regulators (panGWAS odds ratio > 3.7; *P*-value <.001), the complete pathway for three hydrogen cyanide synthase subunits (panGWAS odds ratio > 5.03; *P*-value = .0001), four vitamin B12 transporters (panGWAS odds ratio = 4.31–5.67; *P*-value <.0001), and multidrug transporters (panGWAS odds ratio = 6.67; *P*-value <.000). Additionally, two glycoside hydrolases, a chitinase (panGWAS odds ratio = 5.58; *P*-value <.0001) and a chitodextrinase (panGWAS odds ratio = 4.32; *P*-value <.0001), were also more common in the nonpigmented host-associated genomes. Of the 70 hypothetical proteins, five were predicted to play a role in toxin/antimicrobial systems (panGWAS odds ratio = 4.32; *P*-value <.0001), and three genes were predicted to play a role in antibiotic resistance (panGWAS odds ratio = 5.27–5.57; *P-*value <.0001). Additionally, two genes were predicted to be involved in amino acid transport (panGWAS odds ratio = 4.89–5.56; *P*-value <.0002) and two were predicted by AI gene annotators to contribute to biofilm formation or cellular adhesions (panGWAS odds ratio = 5.15–6.21; *P*-value <.0001). A single gene was predicted to play a role in phospholipid biosynthesis (panGWAS odds ratio = 6.04; *P*-value = .0002). Genes involved in specialized nutrient and amino acid transport systems, fatty acid and lipid biosynthesis, and membrane-associated proteins are typically more common in bacteria that have a symbiotic lifestyle [98]. Gene pathways also suggest that host-associated *Pseudoalteromonas* lineages may be able to utilize alternative carbon sources, such as malate, under aerobic conditions [99, 100]. Additionally, antimicrobial compounds and toxin/antitoxin systems are common in marine invertebrate microbiomes and may help host organisms resist pathogenic infections [101, 102].

**Figure 6:**
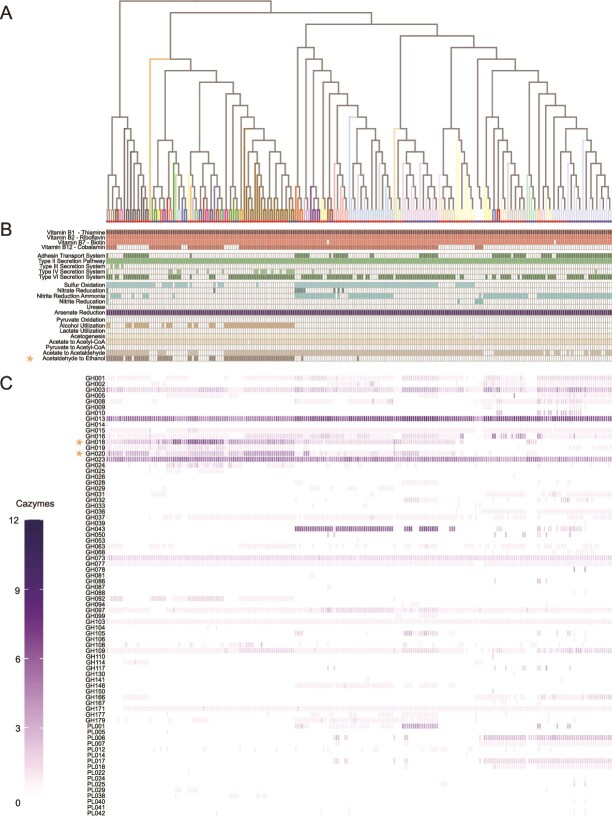
Host-associated *Pseudoalteromonas* lineages are enriched for genes and pathways related to B vitamin production, antimicrobial/antitoxin compounds, breakdown of organic molecules, and host–bacterial interactions. (A) Midpoint-rooted maximum-likelihood phylogenetic tree of the *Pseudoalteromonas* pangenome. The tips indicated whether the isolate was a free-living (blue circle) or host-associated (red circle) strain, and the branch color indicates the phylogroup. (B) A summary of the metabolic and biogeochemical traits, and (C) the heatmap of the abundance of CAZymes predicted by METABOLIC [82]. Orange stars indicate functions that are enriched in host-associated pigmented genomes. Colors from phylogroups are the same as Fig. 2A.

We next used METABOLIC [82] and antiSMASH [84] to predict metabolic pathways, biogeochemical functions, and secondary metabolites across the *Pseudoalteromonas* genomes in our dataset, to identify functions and genes that may facilitate host–microbe interactions (Fig. 6 and Table S4). Several functions that facilitate bacteria−eukaryote interactions were commonly found across the entire pangenome [103–105]. For example, all the *Pseudoalteromonas* genomes in our dataset are predicted to synthesize vitamins B1 (thiamin) and B2 (riboflavin). Most lineages, with the exception of two genomes, are predicted to synthesize vitamin B7 (biotin), and approximately 60% of the genus can synthesize vitamin B12 (cobalamin). Additionally, about 70% of *Pseudoalteromonas* taxa (165 genomes) are predicted to contain a type VI secretion system, which is typically found in ~52% of *Gammaproteobacteria* but is absent in the majority of bacterial phyla [106]. Nearly half (~48%) were predicted to perform sulfur oxidation via sulfur dioxygenases, an essential step in sulfide detoxification. Some functions were conserved across the pangenome, including arsenate reduction, acetogenesis, and type II secretion (Fig. 6).

Only one endopeptidase (C56) was more common in host-associated genomes within the pigmented clade (panGWAS odds ratio = 9.80; *P-*value = .002). In the nonpigmented clade, three functions were more abundant in the host-associated genomes: cellulose degradation (panGWAS odds ratio = 7.23; *P-*value <.001), chitin degradation (panGWAS odds ratio = 7.23; *P-*value <.001), and ethanol fermentation (panGWAS odds ratio = 3.30; *P-*value = .003; Table S4). Three peptidases (S09B, S01A, and C56; panGWAS odds ratio = 3.30–5.60; *P*-value <.01) and two CAZymes, GH18 (a chitinase) and GH20 (hexosaminidase), were also more common in the host-associated genomes (panGWAS odds ratio = 3.92; *P*-value <.001; Table S4). Taken together, our analysis of functional pathways suggests that the bacterial genus *Pseudoalteromonas* may be evolutionarily primed for symbiosis, as most lineages within this genus inherently possess genomic machinery that would provide nutritional benefits for animal hosts (e.g. the nearly universal ability to produce vitamins B1, B2, B7, and the majority of the genus maintaining an additional pathway for vitamin B12). Despite only being found in 50% of *Gammaproteobacteria*, Type II Secretion, which allows for nutrient absorption from the environment and promotes symbiosis in nonpathogenic bacteria [107–109], was universal across the pangenome.

### Localization of *Pseudoalteromonas* near mucus glands in nematodes

**Figure 7:**
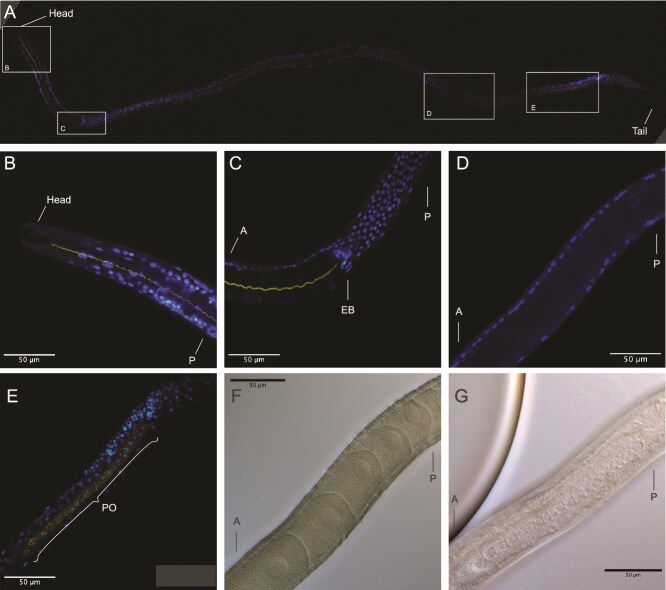
*Pseudoalteromonas* symbionts inhabit a physical niche in the esophagus and ovaries of oncholaimid nematodes. (A) Bacterial biofilms within whole worms were visualized using FISH and found to occur in the esophagus near mucus excretions from the pharyngeal saliva glands, as well as within the underdeveloped ovaries of an adult female Oncholaimidae (genus: *Adoncholaimus*) nematodes. *Pseudoalteromonas* (in yellow) was found in (B) the esophagus of the marine nematode and (C) did not extend past the esophageal bulb (EB). Additionally, *Pseudoalteromonas* and other bacteria (in red) were found in (E, G) the underdeveloped posterior ovary (PO) of the female worm. (D, F) The uninfected anterior ovary was fully developed. In panels (B–G), the A and P indicate the anterior and posterior regions, respectively.

We further investigated the physical location of host-associated *Pseudoalteromonas* bacteria using FISH to visualize symbionts within the body of oncholaimid marine nematodes. Using nematode specimens collected from different seasons and years spanning two habitats (temperate muddy sediments in Tybee Island, GA, and coarse tropical sands in the Florida Keys), we used specific probes to detect *Pseudoalteromonas* [110]. Our FISH results are consistent with previous SEM observations of “rod-shaped” bacteria in the mouth of oncholaimid nematodes isolated from deep-sea hydrothermal vents [111], which is the expected phenotype of *Pseudoalteromonas* bacterial cells. We observed *Pseudoalteromonas* bacteria in the nematode mouth, forming biofilms on the teeth and walls of the buccal cavity (Fig. 7 and Fig. S4), and as biofilms lining the sides of the esophagus down to the esophageal bulb (Fig. 7), which was consistent with biofilm functional pathway prediction from genome analysis (described above). Past the esophageal bulb, host cells were stained blue by DAPI, but there were no positive results for *Pseudoalteromonas* within the nematode gut or intestinal tract (Fig. 7) This suggests that marine nematodes do not digest these symbionts as a food source. In a subset of specimens, additionally found *Pseudoalteromonas* and other bacterial cells colocalized within the underdeveloped posterior ovaries. In these nematodes bacteria were not present in the fully developed anterior ovaries, suggesting that *Pseudoalteromonas* may also be a vertically transmitted pathogen that infects the reproductive organs of marine nematodes (Fig. 7D−G and Fig. S4), as has been suggested for other endosymbiont lineages that are found localized within marine nematode ovaries [112].

## Discussion

Our data broadly support the existence of phylosymbiosis in marine invertebrate microbiomes, as evidenced through pangenomic analyses, host/bacterial phylogenetic topologies, the geographic consistency of symbiosis patterns (e.g. inclusive of Antarctic invertebrates), and FISH imaging, which confirms the formation of host-associated biofilms and identifies a physical niche for *Pseudoalteromonas* symbionts. Our pangenomic study challenges the prior claims that marine invertebrates lack cophylogenetic signals [20], emphasizing the inadequacy of low-resolution 16S rRNA marker gene surveys for robustly assessing marine phylosymbiosis, and demonstrating the need for focused studies and phylogenomic analyses to evaluate evolutionary patterns. In our study of *Pseudoalteromonas*, phylogenetic comparisons of host/symbiont tree topologies exhibited patterns consistent with predictions for complex symbioses involving multiple hosts and bacterial lineages [7]. By using genome-scale data from a single bacterial genus, we found substantial evidence of cophylogenetic signals between *Pseudoalteromonas* and marine invertebrates at the kingdom (Animalia), phylum (Nematoda, Mollusca, and Cnidaria), and family levels (Bivalvia and Faviidae) but weak or no evidence of phylogenetic congruence. Phylogenetic congruence is the most stringent method to assess cophylogeny, but such strict patterns are more likely to emerge in metazoan systems with vertically acquired symbionts, such as terrestrial insects [7, 71]. In marine environments, horizontal transmission is the predominant method of symbiont acquisition [10, 14, 113, 114], which often results in phylogenetic incongruence despite other scenarios that can lead to cophylogenetic signals, such as trait-matching and vicariance events [7, 71]. Trait-based cophylogenetic signals, when the host interacts with bacterial groups that have a specific genomic trait, often cause phylogenetic incongruence, particularly in cases of bacterial groups that undergo substantial host switching [7, 115]. Our phylogenomic and pangenomic evidence indicates that *Pseudoalteromonas* has an open pangenome that is associated with bacteria with a facultative symbiotic lifestyle and large effective population size [116], which can further obscure cophylogenetic signals. In addition, at least five *Pseudoalteromonas* lineages appear to co-exist in marine nematode microbiomes, consistent with the emerging view that intrahost symbiont diversity is more prevalent than previously acknowledged [117, 118]. Our confirmation of cophylogenetic signal between marine invertebrates and *Pseudoalteromonas*, a common “core microbiome” taxon, suggests that host−bacterial codiversification in marine ecosystems is more common than currently understood, and that other core microbiome taxa contributing to phylosymbiosis signals in diverse marine invertebrates [9, 18, 21, 23] should be the target for deeper analyses that leverage whole bacterial genomes.

The host-associated lifestyle of *Pseudoalteromonas* is indicative of a nutritional symbiosis, where bacteria colonize specific physical niches on their animal hosts (e.g. forming biofilms in the nematode mouth and esophagus), outcompete other taxa, and stave off pathogens via production of antimicrobial compounds. In turn, *Pseudoalteromonas* lineages appear to provide their marine invertebrate hosts with food in the form of small organic molecules (simple carbohydrates and protein) as well as essential vitamin B compounds, which animal hosts must obtain exogenously. *Pseudoalteromonas* symbionts exhibit strong parallels with a number of other host−symbiont systems in terrestrial and marine ecosystems. First, the features of host-associated *Pseudoalteromonas* appear to mirror those of another emerging *Gammaproteobacteria* symbionts in marine invertebrates, *Endozoicomonas*. Both taxa demonstrate extracellular aggregations of symbiont bacteria, enrichment of pathways for cycling and transport of carbohydrates and protein, enrichment of vitamin B biosynthesis, and larger genomes with a high degree of genomic plasticity (including a high number of mobile genetic elements), which likely facilitates host switching and lifestyle flexibility in these bacterial genera [42, 119–121]. Pangenomes similar to *Pseudoalteromonas* and *Endozoicomonas*—containing a larger repertoire of accessory and unique genes—are associated with higher rates of horizontal gene transfer, which allows bacteria to rapidly gain and lose genes and increase their genetic diversity compared to closely related strains [122, 123], providing a competitive advantage for facultative symbiosis. Multiple *Endozoicomonas* are often recovered in association with a single coral host, further suggesting that niche partitioning among co-occurring symbiont strains may be typical for these extracellular, horizontally acquired marine invertebrate symbionts [119, 120]. Second, the enrichment of vitamin B biosynthesis and transport pathways in *Pseudoalteromonas* is analogous to terrestrial arthropod symbioses, where bacterial symbionts provide hosts with up to eight B vitamin molecules that supplement the nutritionally poor diet of blood- and sap-feeding host species [104, 124]. Bacterial supplies of B vitamins are known to impact fitness, development, and reproduction in a range of invertebrate taxa [104, 125–128]. For nematodes in particular, bacterial B vitamin production has been shown to enhance predatory behavior known as “surplus killing,” where nematode hosts kill an excess of prey that they do not consume [129]. Thus, genomic pathways enriched in host-associated *Pseudoalteromonas* may directly influence host growth, behavior, and reproduction, which, in turn, provides a direct benefit to extracellular symbionts by increasing the local availability of food and host-associated niches.

We hypothesize that *Pseudoalteromonas* bacteria may be reliant on the mucus of their invertebrate hosts, using mucus as a source of nutrition and/or protective agent that facilitates symbiosis. Invertebrate mucus has ancient evolutionary origins, and mucin-like glycoproteins or domains have been identified in fungi, parasites, and even viruses [130–133]. Animals produce mucus as a physical and chemical barrier that serves as the first line of defense against pathogen infection and disturbance [8, 134, 135], and many marine invertebrates utilize mucus to facilitate locomotion or adhesion, gather prey and detritus [136–138], or enhance reproduction (e.g. as an antimicrobial-infused surface for laying eggs [139]). Furthermore, mucus properties are species-specific in marine invertebrates, exhibiting distinct nutritional profiles (e.g. varying ratios of carbohydrates, proteins, and lipids [139, 140]) and often producing a high number of bioactive and antimicrobial compounds (with the mucus of polychaetes, corals, and sponges often targeted for natural products and drug discovery research [8, 139–142]). In corals, heterotrophic bacteria are known to quickly colonize mucus layers and are capable of growing on mucus as their sole source of carbon [139, 143], and other commensal bacteria such as *Akkermansia*, *Bacteroides*, and *Vibrio* are known to feed on or degrade host mucus using mucolytic enzymes [135, 144]. Experimental work has also shown that the mucus of oncholaimid nematodes promotes the exclusive growth of *Pseudoalteromonas* bacteria, and oncholaimid nematodes in particular are known to excrete copious amounts of mucus from the pharyngeal salivary glands where our FISH images indicated bacterial biofilms were localized [27, 136]. Oncholaimid nematodes are also known to rapidly pump seawater through their intestinal tract [145] and can directly uptake dissolved organic carbon compounds such as glucose and acetate through their intestinal wall [146, 147], suggesting that nematode hosts may rapidly ingest and assimilate bacterial B vitamins and small organic molecules produced by *Pseudoalteromonas* symbiont biofilms. However, the localization of *Pseudoalteromonas* bacteria within stunted female nematode ovaries also hints at more complex host−microbe dynamics, and some strains may exhibit pathogenicity in nematodes similar to previous reports from sponge and coral hosts [47–49].

Physical attachment to the mucus layer of invertebrate hosts is thought to maximize the activity of bioactive substances and provide a competitive advantage for bacteria (e.g. preventing dilution of antimicrobial compounds into the surrounding water [8, 9]). The localization of *Pseudoalteromonas* in the nematode mouth and esophagus may also point toward multiple successional stages of bacterial colonization or species-specific symbioses where the physical host niche varies across different oncholaimid nematodes; however, further experimental work would be needed to confirm these hypotheses. We hypothesize that invertebrate mucus is the specific trait driving the cophylogenetic signals reported in this study, with symbiosis and host switching determined by the molecular composition and antimicrobial properties of invertebrate host species’ mucus. Over evolutionary timescales, invertebrate mucus may have served as a protected microhabitat and consistent source of food, encouraging the establishment of symbiosis and codiversification of invertebrate−bacterial partnerships in marine environments worldwide. Taken together, these findings rapidly advance our understanding of the ecology and evolution of marine invertebrate microbiomes across the Tree of Life. In particular, this study sheds light on the importance of bacterial symbiosis in even the smallest animal phyla, small-bodied metazoa such as nematodes which are numerically abundant and species-rich in benthic habitats, from intertidal beach sands down to the deepest ocean trenches.

In conclusion, this study established that *Pseudoalteromonas* is a symbiont of marine invertebrates with broad host ranges and confirms the existence of phylosymbiosis across three marine invertebrate phyla. The presence of complete biosynthesis pathways of several B vitamins and the ability to degrade complex carbohydrates suggests that *Pseudoalteromonas* may be a nutritional symbiont of marine invertebrates. In exchange, nematode hosts may provide mucus secretions that contain a variety of organic molecules, included amino acids, that could be used as a source of rich nutrients for bacteria. The localization of *Pseudoalteromonas* within developmentally stunted ovaries also suggests that some symbiont strains could also be reproductive manipulators of marine invertebrates; however, future work is needed to confirm these hypotheses.

## Supplementary Material

Supplementary_material_wrag091

## Data Availability

All the genomes used in this study are publicly available in the NCBI RefSeq (Table S1). Raw metagenomic sequences generated by the Georgia Genomics and Bioinformatics Core (GGBC) and the Joint Genomic Institute (JGI, Proposal ID: 505025) were deposited in the NCBI Sequence Read Archive (BioProject: PRJNA1415842) and JGI Genomes Online Database (GOLD Project: Gs0157300), respectively.

## Code availability

All scripts and files required for the reproducibility of all analyses conducted in this study are available via GitHub (https://github.com/BikLab/Pseudoalteromonas).

## References

[1] Lim SJ, Bordenstein SR. An introduction to phylosymbiosis. *Proc Biol Sci* 2020;287:20192900. 10.1098/rspb.2019.2900 32126958 PMC7126058

[2] Sogin EM, Kleiner M, Borowski C et al. Life in the dark: Phylogenetic and physiological diversity of chemosynthetic symbioses. *Ann Rev Microbiol* 2021;75:695–718. 10.1146/annurev-micro-051021-123130 34351792

[3] Osman EO, Weinnig AM. Microbiomes and obligate symbiosis of deep-sea animals. *Annu Rev Anim Biosci* 2022;10:151–76. 10.1146/annurev-animal-081621-112021 34843386

[4] Sunagawa S, Acinas SG, Bork P et al. *Tara* Oceans: towards global ocean ecosystems biology. *Nat Rev Microbiol* 2020;18:428–45. 10.1038/s41579-020-0364-5 32398798

[5] Martínez A, Bonaglia S, Di Domenico M et al. Fundamental questions in meiofauna research highlight how small but ubiquitous animals can improve our understanding of Nature. *Commun Biol* 2025;8:449. 10.1038/s42003-025-07888-1 40097602 PMC11914145

[6] Bik HM, Porazinska DL, Creer S et al. Sequencing our way towards understanding global eukaryotic biodiversity. *Trends Ecol Evol* 2012;27:233–43. 10.1016/j.tree.2011.11.010 22244672 PMC3311718

[7] Dismukes W, Braga MP, Hembry DH et al. Cophylogenetic methods to untangle the evolutionary history of ecological interactions. *Annu Rev Ecol Evol Syst* 2022;53:275–98. 10.1146/annurev-ecolsys-102320-112823

[8] Shnit-Orland M, Kushmaro A. Coral mucus-associated bacteria: a possible first line of defense. *FEMS Microbiol Ecol* 2009;67:371–80. 10.1111/j.1574-6941.2008.00644.x 19161430

[9] O’Brien PA, Webster DJ, Bourne DG et al. Host-microbe coevolution: applying evidence from model systems to complex marine invertebrate holobionts. *MBio* 2019;10:e0224118. 10.1128/mbio.02241-18 PMC6428750 30723123

[10] Russell SL . Transmission mode is associated with environment type and taxa across bacteria-eukaryote symbioses: a systematic review and meta-analysis. *FEMS Microbiol Lett* 2019;366:fnz013. 10.1093/femsle/fnz013 30649338

[11] Moran NA . Symbiosis as an adaptive process and source of phenotypic complexity. *Proc Natl Acad Sci* 2007;104:8627–33. 10.1073/pnas.0611659104 17494762 PMC1876439

[12] McCutcheon JP, Moran NA. Extreme genome reduction in symbiotic bacteria. *Nat Rev Microbiol* 2012;10:13–26. 10.1038/nrmicro2670 22064560

[13] Boscaro V, Kolisko M, Felletti M et al. Parallel genome reduction in symbionts descended from closely related free-living bacteria. *Nat Ecol Evol* 2017;1:1160–7. 10.1038/s41559-017-0237-0 29046583

[14] Breusing C, Genetti M, Russell SL et al. Horizontal transmission enables flexible associations with locally adapted symbiont strains in deep-sea hydrothermal vent symbioses. *Proc Natl Acad Sci* 2022;119:e2115608119. 10.1073/pnas.2115608119 35349333 PMC9168483

[15] Morrow KM, Bourne DG, Humphrey C et al. Natural volcanic CO_2_ seeps reveal future trajectories for host-microbial associations in corals and sponges. *ISME J* 2015;9:894–908. 10.1038/ismej.2014.188 25325380 PMC4817704

[16] Neave MJ, Rachmawati R, Xun L et al. Differential specificity between closely related corals and abundant *Endozoicomonas* endosymbionts across global scales. *ISME J* 2016;11:186–200. 10.1038/ismej.2016.95 27392086 PMC5335547

[17] Holt CC, Dhaliwal S, Na I et al. Spatial compartmentalisation of bacteria in phoronid microbiomes. *Sci Rep* 2023;13:18612. 10.1038/s41598-023-45652-9 37903823 PMC10616082

[18] Leasi F, Eckert EM, Norenburg JL et al. Microbiota associated with *Ototyphlonemertes* species (Nemertea, Hoplonemertea, Monostilifera, Ototyphlonemertidae) reveal evidence of phylosymbiosis. *Ecol Evol* 2024;14:e70471. 10.1002/ece3.70471 39629175 PMC11612514

[19] M MC, Goulet TL, Jackson CR et al. Systematic review of cnidarian microbiomes reveals insights into the structure, specificity, and fidelity of marine associations. *Nat Commun* 2023;14:4899. 10.1038/s41467-023-39876-6 37580316 PMC10425419

[20] Boscaro V, Holt CC, Van Steenkiste NWL et al. Microbiomes of microscopic marine invertebrates do not reveal signatures of phylosymbiosis. *Nat Microbiol* 2022;7:810–9. 10.1038/s41564-022-01125-9 35618773

[21] O’Brien PA, Tan S, Yang C et al. Diverse coral reef invertebrates exhibit patterns of phylosymbiosis. *ISME J* 2020;14:2211–22. 10.1038/s41396-020-0671-x 32444811 PMC7608455

[22] Prioux C, Ferrier-Pages C, Deter J et al. Insights into the occurrence of phylosymbiosis and co-phylogeny in the holobionts of octocorals from the Mediterranean Sea and Red Sea. *Anim Microbiome* 2024;6:62. 10.1186/s42523-024-00351-2 39497183 PMC11533408

[23] Tsang CTT, Hui TKL, Chung NM et al. Comparative analysis of gut microbiome of mangrove brachyuran crabs revealed patterns of phylosymbiosis and codiversification. *Mol Ecol* 2024;33:e17377. 10.1111/mec.17377 38713089

[24] Pereira TJ, De Santiago A, Schuelke T et al. The impact of intragenomic rRNA variation on metabarcoding-derived diversity estimates: A case study from marine nematodes. *Environ DNA* 2020;2:519–34. 10.1002/edn3.77

[25] Bairoliya S, Koh Zhi Xiang J, Cao B. Extracellular DNA in environmental samples: Occurrence, extraction, quantification, and impact on microbial biodiversity assessment. *Appl Environ Microbiol* 2022;88:e0184521. 10.1128/aem.01845-21 34818108 PMC8824265

[26] Bellec L, Bonavita MAC, Hourdez S et al. Chemosynthetic ectosymbionts associated with a shallow-water marine nematode. *Sci Rep* 2019;9:7019. 10.1038/s41598-019-43517-8 31065037 PMC6505526

[27] Moens T, Santos GAP, Thompson F et al. Do nematode mucus secretions affect bacterial growth? *Aquat Microb Ecol* 2005;40:77–83. 10.3354/ame040077

[28] Pereira TJ, Walters TL, . et al. The microbiome of the pelagic tunicate Dolioletta gegenbauri: A potential link between the grazing and microbial food web. Mol Ecol 2023;32:6564–79. 10.1111/mec.16668 35989550

[29] Ohdera A, Attarwala K, Wu V et al. Comparative genomic insights into bacterial induction of larval settlement and metamorphosis in the upside-down jellyfish *Cassiopea*. *mSphere* 2023;8:e0031522. 10.1128/msphere.00315-22 37154768 PMC10286705

[30] Delgadillo-Ordoñez N, Garcias-Bonet N, Raimundo I et al. Probiotics reshape the coral microbiome in situ without detectable off-target effects in the surrounding environment. *Commun Biol* 2024;7:434. 10.1038/s42003-024-06135-3 38594357 PMC11004148

[31] Osman EO, Suggett DJ, Voolstra CR et al. Coral microbiome composition along the northern Red Sea suggests high plasticity of bacterial and specificity of endosymbiotic dinoflagellate communities. *Microbiome* 2020;8:8. 10.1186/s40168-019-0776-5 32008576 PMC6996193

[32] Williams SE, Varliero G, Lurgi M et al. Diversity and structure of the deep-sea sponge microbiome in the equatorial Atlantic Ocean. *Microbiology* 2024;170:e001478. 10.1099/mic.0.001478 PMC11286294 39073401

[33] Shan X, Li K, Stadler P et al. Microbiome determinants of productivity in aquaculture of whiteleg shrimp. *Appl Environ Microbiol* 2025;91:e0242024. 10.1128/aem.02420-24 40231846 PMC12094023

[34] Zhu Y-T, Liang X, Liu T-T et al. The mussel larvae microbiome changes in response to a temperature rise. *Front Mar Sci* 2024;11:e1367608. 10.3389/fmars.2024.1367608

[35] Seibold A, Wichels A, Schütt C. Diversity of endocytic bacteria in the dinoflagellate *Noctiluca scintillans*. *Aquat Microb Ecol* 2001;25:229–35. 10.3354/ame025229

[36] Salazar G, Cornejo-Castillo FM, Benitez-Barrios V et al. Global diversity and biogeography of deep-sea pelagic prokaryotes. *ISME J* 2015;10:596–608. 10.1038/ismej.2015.137 26251871 PMC4817678

[37] Wietz M, Gram L, Jørgenson B et al. Latitudinal patterns in the abundance of major marine bacterioplankton groups. *Aquat Microb Ecol* 2010;61:179–89. 10.3354/ame01443

[38] Yaphe W . The use of agarase from Pseudomonas atlantica in the identification of agar in marine algae (Rhodophyceae). *Can J Microbiol* 1957;3:987–93. 10.1139/m57-109 13489548

[39] Atencio LA, Dal Grande F, Young GO et al. Antimicrobial-producing *Pseudoalteromonas* from the marine environment of Panama shows a high phylogenetic diversity and clonal structure. *J Basic Microbiol* 2018;58:747–69. 10.1002/jobm.201800087 29938809

[40] Bell AG, McMurtie J, Bolaños LM et al. Influence of host phylogeny and water physicochemistry on microbial assemblages of the fish skin microbiome. *FEMS Microbiol Ecol* 2024;100:fiae021. 10.1093/femsec/fiae021 PMC10903987 38366921

[41] Adnani N, Rajski SR, Bugni TS. Symbiosis-inspired approaches to antibiotic discovery. *Nat Prod Rep* 2017;34:784–814. 10.1039/C7NP00009J 28561849 PMC5555300

[42] Bosi E, Fondi M, Orlandini V et al. The pangenome of (Antarctic) *Pseudoalteromonas* bacteria: evolutionary and functional insights. *BMC Genomics* 2017;18:93. 10.1186/s12864-016-3382-y 28095778 PMC5240218

[43] Ma Y, Sun F, Zhang C et al. Effects of *Pseudoalteromonas* sp. BC228 on digestive enzyme activity and immune response of juvenile sea cucumber (*Apostichopus japonicus*). *J Ocean Univ China* 2014;13:1061–6. 10.1007/s11802-014-2340-z

[44] Ericson CF, Eisenstein F, Medeiros JM et al. A contractile injection system stimulates tubeworm metamorphosis by translocating a proteinaceous effector. *elife* 2019;8:e46845. 10.7554/eLife.46845 31526475 PMC6748791

[45] Malter KE, Dunbar TL, Westin C et al. A bacterial membrane-disrupting protein stimulates animal metamorphosis. *MBio* 2025;16:e0357324. 10.1128/mbio.03573-24 39727418 PMC11796346

[46] Peng LH, Liang X, Xu JK et al. Monospecific biofilms of *Pseudoalteromonas* promote larval settlement and metamorphosis of *Mytilus coruscus*. *Sci Rep* 2020;10:2577. 10.1038/s41598-020-59506-1 32054934 PMC7018757

[47] Beurmann S, Ushijima B, Videau P et al. *Pseudoalteromonas piratica* strain OCN003 is a coral pathogen that causes a switch from chronic to acute *Montipora* white syndrome in *Montipora capitata*. *PLoS One* 2017;12:e0188319. 10.1371/journal.pone.0188319 29145488 PMC5690655

[48] Choudhury JD. et al. The pathogen of the great barrier reef sponge *Rhopaloeides odorabile* is a new strain of *Pseudoalteromonas agarivorans* containing abundant and diverse virulence-related genes. *Mar Biotechnol* 2015;17:463–78. 10.1007/s10126-015-9627-y 25837832

[49] Romanenko LA, Zhukova NV, Rohde M et al. *Pseudoalteromonas agarivorans* sp. nov., a novel marine agarolytic bacterium. *Int J Syst Evol Microbiol* 2003;53:125–31. 10.1099/ijs.0.02234-0 12656163

[50] De Santiago A, Barnes SJ, Pereira TJ et al. Complete genome sequences of two *Pseudoalteromonas undina* strains isolated from a marine nematode (Oncholaimidae) collected at Tybee Island. *Microbiol Resour Announc* 2025;14:e0041925. 10.1128/mra.00419-25 40709927 PMC12352060

[51] Schuelke T, Pereira TJ, Hardy SM et al. Nematode-associated microbial taxa do not correlate with host phylogeny, geographic region or feeding morphology in marine sediment habitats. *Mol Ecol* 2018;27:1930–51. 10.1111/mec.14539 29600535

[52] R. Danovaro Methods for the study of deep-sea sediments, their functioning and biodiversity (1st ed.). Florida: CRC Press 2009. 10.1201/9781439811382

[53] WoRMS Editorial Board . World Register of Marine Species (WoRMS). https://www.marinespecies.org Belgium: Flanders Marine Institute (VLIZ). (January 2025, date last accessed), 10.14284/170.

[54] Platt HM, Warwick RM. Free-living marine nematodes Part 1: British enoplids. In: Kermack and Barnes (eds.), Synopses of the British Fauna. London: Cambridge University Press, 1983.

[55] Platt HM, Warwick RM, Furstenberg JP. Free-living Marine Nematodes. Part 1 British Enoplids. *S Afr J Zool* 1985;20:177–7.

[56] Platt HM, Warwick RM. Free-living marine nematodes Part 2: British chromadorids. In: Kermack and Barnes (eds.), Synopses of the British fauna. Leiden: Brill, 1988.

[57] Warwick RM, Platt HM, Somerfield PJ. Free-living marine nematodes Part 3: Monhysterids. In: Barnes and Crothers (eds.), Synopses of the British Fauna. Shrewsbury: Fields Studies Council, 1998.

[58] Derycke S, De Meester N, Rigaux A et al. Coexisting cryptic species of the *Litoditis marina* complex (Nematoda) show differential resource use and have distinct microbiomes with high intraspecific variability. *Mol Ecol* 2016;25:2093–110. 10.1111/mec.13597 26929004

[59] Yoder M, De Ley IT, King TW et al. DESS: a versatile solution for preserving morphology and extractable DNA of nematodes. *Nematology* 2006;8:367–76. 10.1163/156854106778493448

[60] Seemann T . Prokka: rapid prokaryotic genome annotation. *Bioinformatics* 2014;30:2068–9. 10.1093/bioinformatics/btu153 24642063

[61] The UniProt Consortium . UniProt: the universal protein knowledgebase in 2023. *Nucleic Acids Res* 2023;51:532–1. 10.1093/nar/gkac1052 PMC9825514 36408920

[62] Bayliss SC, Thorpe HA, Coyle NM et al. PIRATE: A fast and scalable pangenomics toolbox for clustering diverged orthologues in bacteria. *Gigascience* 2019;8:giz119. 10.1093/gigascience/giz119 PMC6785682 31598686

[63] Katoh K, Standley DM. MAFFT multiple sequence alignment software version 7: improvements in performance and usability. *Mol Biol Evol* 2013;30:772–80. 10.1093/molbev/mst010 23329690 PMC3603318

[64] Stamatakis A . RAxML version 8: a tool for phylogenetic analysis and post-analysis of large phylogenies. *Bioinformatics* 2014;30:1312–3. 10.1093/bioinformatics/btu033 24451623 PMC3998144

[65] Olm MR, Brown CT, Brooks B et al. dRep: a tool for fast and accurate genomic comparisons that enables improved genome recovery from metagenomes through de-replication. *ISME J* 2017;11:2864–8. 10.1038/ismej.2017.126 28742071 PMC5702732

[66] Jain C, Rodriguez-R LM, Phillippy AM et al. High throughput ANI analysis of 90K prokaryotic genomes reveals clear species boundaries. *Nat Commun* 2018;9:5114. 10.1038/s41467-018-07641-9 30504855 PMC6269478

[67] Harling-Lee JD, Gorzynski J, Yebra G et al. A graph-based approach for the visualisation and analysis of bacterial pangenomes. *BMC Bioinformatics* 2022;23:416. 10.1186/s12859-022-04898-2 36209064 PMC9548110

[68] Freeman TC, Horsewell S, Patir A et al. Graphia: A platform for the graph-based visualisation and analysis of high dimensional data. *PLoS Comput Biol* 2022;18:e1010310. 10.1371/journal.pcbi.1010310 35877685 PMC9352203

[69] Gregor R, Probst M, Eyal S et al. Mammalian gut metabolomes mirror microbiome composition and host phylogeny. *ISME J* 2022;16:1262–74. 10.1038/s41396-021-01152-0 34903850 PMC9038745

[70] Kumar S, Suleski M, Craig JM et al. TimeTree 5: An expanded resource for species divergence times. *Mol Biol Evol* 2022;39:msac174. 10.1093/molbev/msac174 PMC9400175 35932227

[71] Perez-Lamarque B, Morlon H. Distinguishing cophylogenetic signal from phylogenetic congruence clarifies the interplay between evolutionary history and species interactions. *Syst Biol* 2024;73:613–22. 10.1093/sysbio/syae013 38477631

[72] Balbuena JA, Míguez-Lozano R, Blasco-Costa I. PACo: A novel procrustes application to cophylogenetic analysis. *PLoS One* 2013;8:e61048. 10.1371/journal.pone.0061048 23580325 PMC3620278

[73] Böcker S, Canzar S, Klau GW. The Generalized Robinson-Foulds Metric. *Algorithms in Bioinformatics* 2013;8126:156–69. 10.1007/978-3-642-40453-5_13

[74] Smith MR . Information theoretic generalized Robinson–Foulds metrics for comparing phylogenetic trees. *Bioinformatics* 2020;36:5007–13. 32619004 10.1093/bioinformatics/btaa614

[75] Stone EA, Sidow A. Constructing a meaningful evolutionary average at the phylogenetic center of mass. *BMC Bioinformatics* 2007;8:222. 10.1186/1471-2105-8-222 PMC1919398 17594490

[76] Andrews S . FastQC: a quality control tool for high throughput sequence data, United Kingdom: Babraham Institute. https://www.bioinformatics.babraham.ac.uk/projects/fastqc/

[77] Ewels P, Magnusson M, Lundin S et al. MultiQC: summarize analysis results for multiple tools and samples in a single report. *Bioinformatics* 2016;32:3047–8. 10.1093/bioinformatics/btw354 27312411 PMC5039924

[78] Bolger AM, Lohse M, Usadel B. Trimmomatic: a flexible trimmer for Illumina sequence data. *Bioinformatics* 2014;30:2114–20. 10.1093/bioinformatics/btu170 24695404 PMC4103590

[79] Wood DE, Lu J, Langmead B. Improved metagenomic analysis with Kraken 2. *Genome Biol* 2019;20:257. 10.1186/s13059-019-1891-0 31779668 PMC6883579

[80] Kojima CY, Getz EW, Thrash JC. RRAP: RPKM recruitment analysis pipeline. *Microbiol Resour Announc* 2022;11:e0064422. 10.1128/mra.00644-22 35993706 PMC9476942

[81] Yu G, Smith DK, Zhu H et al. GGTREE: An R package for visualization and annotation of phylogenetic trees with their covariates and other associated data. *Methods Ecol Evol* 2017;8:28–36. 10.1111/2041-210X.12628

[82] Zhou Z, Tran PQ, Breister AM et al. METABOLIC: high-throughput profiling of microbial genomes for functional traits, metabolism, biogeochemistry, and community-scale functional networks. *Microbiome* 2022;10:33. 10.1186/s40168-021-01213-8 35172890 PMC8851854

[83] Aramaki T, Blanc-Mathieu R, Endo H et al. KofamKOALA: KEGG Ortholog assignment based on profile HMM and adaptive score threshold. *Bioinformatics* 2020;36:2251–2. 10.1093/bioinformatics/btz859 31742321 PMC7141845

[84] Blin K, Shaw S, Augustijn HA et al. antiSMASH 7.0: new and improved predictions for detection, regulation, chemical structures and visualisation. *Nucleic Acids Res* 2023;51:W46–50. 10.1093/nar/gkad344 37140036 PMC10320115

[85] Roder T, Pimentel G, Fuchsmann P et al. Scoary2: rapid association of phenotypic multi-omics data with microbial pan-genomes. *Genome Biol* 2024;25:93. 10.1186/s13059-024-03233-7 38605417 PMC11007987

[86] Jha N, Kravitz J, West-Roberts J et al. Gaia: An AI-enabled genomic context-aware platform for protein sequence annotation. *Sci Adv* 2025;11:eadv5109. 10.1126/sciadv.adv5109 40540578 PMC12180486

[87] Amann RI, Binder BJ, Olson RJ et al. Combination of 16S rRNA-targeted oligonucleotide probes with flow cytometry for analyzing mixed microbial populations. *Appl Environ Microbiol* 1990;56:1919–25. 10.1128/aem.56.6.1919-1925.1990 2200342 PMC184531

[88] Daims H, Brühl A, Amann R et al. The domain-specific probe EUB338 is insufficient for the detection of all Bacteria: development and evaluation of a more comprehensive probe set. *Syst Appl Microbiol* 1999;22:434–44. 10.1016/S0723-2020(99)80053-8 10553296

[89] Greuter D, Loy A, Horn M et al. probeBase-an online resource for rRNA-targeted oligonucleotide probes and primers: new features 2016. *Nucleic Acids Res* 2016;44:D586–9. 10.1093/nar/gkv1232 26586809 PMC4702872

[90] Wallner G, Amann R, Beisker W. Optimizing fluorescent in situ hybridization with rRNA-targeted oligonucleotide probes for flow cytometric identification of microorganisms. *Cytometry* 1993;14:136–43. 10.1002/cyto.990140205 7679962

[91] Henson MW, Lanclos VC, Pitre DM et al. Expanding the diversity of bacterioplankton isolates and modeling isolation efficacy with large-scale dilution-to-extinction cultivation. *Appl Environ Microbiol* 2020;86:e00943–20. 10.1128/AEM.00943-20 32561583 PMC7440811

[92] Sonnenberg CB, Haugen P. The *Pseudoalteromonas* multipartite genome: distribution and expression of pangene categories, and a hypothesis for the origin and evolution of the chromid. *G3* 2021;11:256. 10.1093/g3journal/jkab256 PMC8496264 34544144

[93] Wang J, Li P, Di X et al. Phylogenomic analysis uncovers an unexpected capacity for the biosynthesis of secondary metabolites in *Pseudoalteromonas*. *Eur J Med Chem* 2024;279:116840. 10.1016/j.ejmech.2024.116840 39244863

[94] Barh D, Soares S, Tiwari S et al. Pan-genomics: Applications, challenges, and future prospects. United Kingdom: Academic Press, 2020. 10.1016/C2018-0-00570-8

[95] Martino ME, Bayjanov JR, Caffrey BE et al. Nomadic lifestyle of *Lactobacillus plantarum* revealed by comparative genomics of 54 strains isolated from different habitats. *Environ Microbiol* 2016;18:4974–89. 10.1111/1462-2920.13455 27422487

[96] Mahajan S, Agashe D. Evolutionary jumps in bacterial GC content. *G3* 2022;12jkac:108. 10.1093/g3journal/jkac108 PMC9339322 35579351

[97] Brooks AW, Kohl KD, Brucker RM et al. Phylosymbiosis: Relationships and functional effects of microbial communities across host evolutionary history. *PLoS Biol* 2016;14:e2000225. 10.1371/journal.pbio.2000225 27861590 PMC5115861

[98] Villada JC, Yumary M, Vasquez GS et al. A genomic catalog of Earth’s bacterial and archaeal symbionts. *bioRxiv* 2025;2025.05.29.656868. 10.1101/2025.05.29.656868 42675165

[99] Xiong L, Chan E, Teng JLL et al. Malate-dependent carbon utilization enhances central metabolism and contributes to biological fitness of *Laribacter hongkongensis* via CRP regulation. *Front Microbiol* 2019;10:1991. 10.3389/fmicb.2019.01991 31555230 PMC6722228

[100] Lukas H, Reimann J, Kim OB et al. Regulation of aerobic and anaerobic D-malate metabolism of *Escherichia coli* by the LysR-type regulator DmlR (YeaT). *J Bacteriol* 2010;192:2503–11. 10.1128/jb.01665-09 20233924 PMC2863561

[101] Pereira LB, Palermo BRZ, Carlos C et al. Diversity and antimicrobial activity of bacteria isolated from different Brazilian coral species. *FEMS Microbiol Lett* 2017;364:fnx164. 10.1093/femsle/fnx164 28873945

[102] Chen L, Wang XY, Liu RZ et al. Culturable microorganisms associated with sea cucumbers and microbial natural products. *Mar Drugs* 2021;19:461. 10.3390/md19080461 34436300 PMC8400260

[103] Sultana S, Bruns S, Wilkes H et al. Vitamin B12 is not shared by all marine prototrophic bacteria with their environment. *ISME J* 2023;17:836–45. 10.1038/s41396-023-01391-3 36914732 PMC10203341

[104] Douglas AE . The B vitamin nutrition of insects: the contributions of diet, microbiome and horizontally acquired genes. *Curr Opin Insect Sci* 2017;23:65–9. 10.1016/j.cois.2017.07.012 29129284

[105] EVS M, Lariviere PJ, Jones KR et al. Type VI secretion systems promote intraspecific competition and host interactions in a bee gut symbiont. *Proc Natl Acad Sci* 2024;121:e2414882121. 10.1073/pnas.2414882121 39441627 PMC11536156

[106] Abby SS, Cury J, Guglielmini J et al. Identification of protein secretion systems in bacterial genomes. *Sci Rep* 2016;6:23080. 10.1038/srep23080 26979785 PMC4793230

[107] Tseng TT, Tyler BM, Setubal JC. Protein secretion systems in bacterial-host associations, and their description in the Gene Ontology. *BMC Microbiol* 2009;9, 9 Suppl 1:S2. 10.1186/1471-2180-9-S1-S2 19278550 PMC2654662

[108] Jani AJ, Cotter PA. Type VI secretion: not just for pathogenesis anymore. *Cell Host Microbe* 2010;8:2–6. 10.1016/j.chom.2010.06.012 External Link 20638635 PMC2913581

[109] Cianciotto NP, White RC. Expanding role of type II secretion in bacterial pathogenesis and beyond. *Infect Immun* 2017;85:e0001417. 10.1128/IAI.00014-17 PMC5400843 28264910

[110] Eilers H. et al. Culturability and in situ abundance of pelagic bacteria from the North Sea. *Appl Environ Microbiol* 2000;66:3044–51. 10.1128/iai.00014-17 10877804 PMC92109

[111] Bellec L, Cambon-Bonavita MA, Cueff-Gauchard V et al. A nematode of the Mid-Atlantic Ridge hydrothermal vents harbors a possible symbiotic relationship. *Front Microbiol* 2018;66:2246 10.3389/fmicb.2018.02246. PMC6159746 30294317

[112] Hagenbeek A, Masukagami Y, Palanichamy P et al. A Midichloriaceae endosymbiont of terrestrial arthropods found as an endosymbiont in a marine nematode. *BioRxiv* 2025; 2025.07.30.667539. 10.1101/2025.07.30.667539

[113] Hauer MA, Breusing C, Trembath-Reichert et al. Geography, not lifestyle, explains the population structure of free-living and host-associated deep-sea hydrothermal vent snail symbionts. *Microbiome* 2023;11:106. 10.1186/s40168-023-01493-2 37189129 PMC10186799

[114] Bright M, Bulgheresi S. A complex journey: transmission of microbial symbionts. *Nat Rev Microbiol* 2010;8:218–30. 10.1038/nrmicro2262 20157340 PMC2967712

[115] Russo L, Miller AD, Tooker J et al. Quantitative evolutionary patterns in bipartite networks: Vicariance, phylogenetic tracking or diffuse co-evolution? *Methods Ecol Evol* 2018;9:761–72. 10.1111/2041-210X.12914

[116] Dewar AE, Hao C, Belcher LJ et al. Bacterial lifestyle shapes pangenomes. *Proc Natl Acad Sci* 2024;121:e2320170121. 10.1073/pnas.2320170121 38743630 PMC11126918

[117] Ansorge R, Romano S, Sayavedro L et al. Functional diversity enables multiple symbiont strains to coexist in deep-sea mussels. *Nat Microbiol* 2019;4:2487–97. 10.1038/s41564-019-0572-9 31611646

[118] Gould AL, Donohoo SA, Román ED et al. Strain-level diversity of symbiont communities between individuals and populations of a bioluminescent fish. *ISME J* 2023;17:2362–9. 10.1038/s41396-023-01550-6 37891426 PMC10689835

[119] Neave MJ, Apprill A, Ferrier-Pagès et al. Diversity and function of prevalent symbiotic marine bacteria in the genus *Endozoicomonas*. *Appl Microbiol Biotechnol* 2016;100:8315–24. 10.1007/s00253-016-7777-0 27557714 PMC5018254

[120] Neave MJ, Michell C, Apprill A et al. *Endozoicomonas* genomes reveal functional adaptation and plasticity in bacterial strains symbiotically associated with diverse marine hosts. *Sci Rep* 2017;7:40579. 10.1038/srep40579 28094347 PMC5240137

[121] Pogoreutz C, Oakley CA, Rädecker N et al. Coral holobiont cues prime *Endozoicomonas* for a symbiotic lifestyle. *ISME J* 2022;16:1883–95. 10.1038/s41396-022-01226-7 35444262 PMC9296628

[122] McInerney JO, McNally A, O’Connell MJ. Why prokaryotes have pangenomes. *Nature* *Microbiology* 2017;2:17040. 10.1038/nmicrobiol.2017.40 28350002

[123] Lim SL, Chin CH, Chiou YJ et al. Unveiling Unusual Ecofunctional Traits of Endozoicomonas Species Through Comprehensive Comparative Genomics. *Environ Microbiol.* 27:e70191. 10.1111/1462-2920.70191 41101942

[124] Buysse M, Floriano AM, Gottlieb Y et al. A dual endosymbiosis supports nutritional adaptation to hematophagy in the invasive tick *Hyalomma marginatum*. *elife* 2021;10:e72747. 10.7554/eLife.72747 34951405 PMC8709577

[125] Haçariz O, Viau C, Karimian F et al. The symbiotic relationship between Caenorhabditis elegans and members of its microbiome contributes to worm fitness and lifespan extension. *BMC Genomics* 2021;22:364. 10.1186/s12864-021-07695-y 34011272 PMC8136213

[126] Feng M, Gao B, Ruiz D et al. Bacterial vitamin B6 is required for post-embryonic development in *C. elegans*. *Commun Biol* 2024;7:367. 10.1038/s42003-024-05992-2 38532074 PMC10966028

[127] Hickin ML, Kakumanu ML, Schal C. Effects of *Wolbachia* elimination and B-vitamin supplementation on bed bug development and reproduction. *Sci Rep* 2022;12:10270. 10.1038/s41598-022-14505-2 35715692 PMC9205976

[128] Croft MT, Lawrence A, Raux-Deery E et al. Algae acquire vitamin B12 through a symbiotic relationship with bacteria. *Nature* 2005;438:90–3. 10.1038/nature04056 16267554

[129] Akduman N, Lightfoot JW, Röseler W et al. Bacterial vitamin B12 production enhances nematode predatory behavior. *ISME J* 2020;14:1494–507. 10.1038/s41396-020-0626-2 32152389 PMC7242318

[130] Freire T, Casaravilla C, Carmona C et al. Mucin-type O-glycosylation in *Fasciola hepatica*: characterisation of carcinoma-associated Tn and sialyl-Tn antigens and evaluation of UDP-GalNAc:polypeptide N-acetylgalactosaminyltransferase activity. *Int J Parasitol* 2003;33:47–56. 10.1016/S0020-7519(02)00231-X 12547345

[131] Wang F, Metcalf T, van der Wel H et al. Initiation of mucin-type O-glycosylation in *dictyostelium* is homologous to the corresponding step in animals and is important for spore coat function. *J Biol Chem* 2003;278:51395–407. 10.1074/jbc.M308756200 14551185

[132] Buscaglia CA, Campo VA, Frasch ACC et al. *Trypanosoma cruzi* surface mucins: host-dependent coat diversity. *Nat Rev Microbiol* 2006;4:229–36. 10.1038/nrmicro1351 16489349

[133] Lang T, Hansson GC, Samuelsson T. Gel-forming mucins appeared early in metazoan evolution. *Proc Natl Acad Sci* 2007;104:16209–14. 10.1073/pnas.0705984104 17911254 PMC2042186

[134] Glasl B, Herndl GJ, Frade PR. The microbiome of coral surface mucus has a key role in mediating holobiont health and survival upon disturbance. *ISME J* 2016;10:2280–92. 10.1038/ismej.2016.9 26953605 PMC4989324

[135] Sheng YH, Hasnain SZ. Mucus and mucins: The underappreciated host defence system. *Front Cell Infect Microbiol* 2022;12:856962. 10.3389/fcimb.2022.856962 35774401 PMC9238349

[136] Riemann F, Schrage M. The mucus-trap hypothesis on feeding of aquatic nematodes and implications for biodegradation and sediment texture. *Oecologia* 1978;34:75–88. 10.1007/BF00346242 28309389

[137] Riemann F, Helmke E. Symbiotic relations of sediment-agglutinating nematodes and bacteria in detrital habitats: The enzyme-sharing concept. *Mar Ecol* 2002;23:93–113. 10.1046/j.1439-0485.2002.02765.x

[138] Wilden B, Madji N, Kuhlicke U et al. Flatworm mucus as the base of a food web. *BMC Ecol* 2019;19:15. 10.1186/s12898-019-0231-2 30925873 PMC6441204

[139] Stabili L . The mucus of marine invertebrates. In: Trincone, E, Enzymatic Technologies for Marine Polysaccharides. Boca Raton: CRC Press, 2019, 151–62.

[140] Stabili L, Licciano M, Giangrande A et al. First insight on the mucus of the annelid *Myxicola infundibulum* (Polychaeta, Sabellidae) as a potential prospect for drug discovery. *Mar Drugs* 2019;17:396. 10.3390/md17070396 31284386 PMC6669576

[141] Varijakzhan D, Loh JY, Yap WS et al. Bioactive compounds from marine sponges: Fundamentals and applications. *Mar Drugs* 2021;19:246. 10.3390/md19050246 33925365 PMC8146879

[142] Li P, Lu H, Zhang Y et al. The natural products discovered in marine sponge-associated microorganisms: structures, activities, and mining strategy. *Front Mar Sci* 2023;10:1191858. 10.3389/fmars.2023.1191858

[143] H. Pascal. Bacterial utilization of mucus on the coral reefs of Aqaba (Red Sea). In: Gomez, Edgardo D. et al (eds), *Proc 4th Int Coral Symp*. Philippines: Marine Sciences Center, University of the Philippines. 1982:1:669–77.

[144] Crowther RS, Roomi NW, Fahim REF et al. *Vibrio cholerae* metalloproteinase degrades intestinal mucin and facilitates enterotoxin-induced secretion from rat intestine. *Biochim Biophys Acta Gen Subj* 1987;924:393–402. 10.1016/0304-4165(87)90153-X 3297167

[145] Moens T, Verbeeck L, Vincx M. Feeding biology of a predatory and a facultatively predatory nematode (*Enoploides longispiculosus* and *Adoncholaimus fuscus*). *Mar Biol* 1999;134:585–93. 10.1007/s002270050573

[146] Chia FS, Warwick RM. Assimilation of labelled glucose from seawater by marine nematodes. *Nature* 1969;224:720–1. 10.1038/224720a0

[147] Riemann F, Ernst W, Ernst R. Acetate uptake from ambient water by the free-living marine nematode *Adoncholaimus thalassophygas*. *Mar Biol* 1990;104:453–7. 10.1007/BF01314349

