# Supplementary material for: Pseudoalteromonas is a symbiont of marine invertebrates that exhibits broad patterns of phylosymbiosis
Source: ISME J. 2026 Apr 20;20(1):wrag091. doi: 10.1093/ismejo/wrag091 (PMC13245728; doi:10.1093/ismejo/wrag091)
Supplement: Supplementary_material_wrag091 [file supplementary_material_wrag091.zip › 2026-04-11-supplementary-material.docx]

**Supplemental Material**

**Fig. S1: Average nucleotide identity reveals a higher diversity within the genus Pseudoalteromonas than previously estimated.** A higher fastANI score correlates with high genetic similarity between clades. A minimum of 95% fastANI was used to place bacterial isolates in the same phylogroup.

**Fig. S2. Additional evidence for cophylogenetic signals between *Pseudoalteromonas* and marine invertebrates.**

Phylogenetic trees of *Pseudoalteromonas* (top) and the hosts (bottom) were used to test for cophylogeny across the Kingdom (Animalia). Topological trees (top) of *Pseudoalteromonas* were constructed with RAxML [64] using the General Time Reversible (GTR) model with GAMMA-distributed rates across sites and 100 bootstraps. Host cladograms (bottom) were generated via TimeTree [70]. Cophylogeny signal was tested using PACo, and phylogenetic congruence was tested using the Generalized Robinson-Foulds metric (**Table 1**).

**Fig. S3: Host-associated genomes contain distinct sets of genes that enable host-bacterial interactions.** A) Midrooted phylogenetic tree of the *Pseudoalteromonas* pangenome. The tips indicate whether the isolate was a free-living (blue circle) or host-associated (red circle) strain, and the branch color indicates the phylogroup. B) Summary of the accessory genes that were more common in the host-associated pigmented (orange) or host-associated nonpigmented (purple) bacterial genomes. The results of the SCOARY2 panGWAS and a summary of the gene products can be found in **Table S3**.

**Fig. S4: FISH of the microbiomes of Oncholaimidae nematodes collected from Tybee Island, GA.** FISH of the head (A-E) and posterior ovary (F-J) of an oncholaimid nematode. In Blue (A,F), DAPI-stained host cells. Eubacterial probes are stained red (B,G), whereas Pseudoalteromonas-specific probes are yellow (C, H). Green indicates nonsense probes that are staining the host cells, but none of the bacterial cells (D,I). Merged images of the DAPI, bacterial, and *Pseudoalteromonas*-specific probes.

**Table S1: Summary of the genomes included in this study.** The lifestyle of the isolated strain (host-associated vs. free-living), the phyla of the host it was isolated from, the completeness and contamination (based on CheckM), and the AccessionID. The two genomes isolated from marine nematodes in this study are highlighted in yellow.

**Table S2: Summary of single-worm metagenomic samples used to identify the prevalence of *Pseudoalteromonas* in the microbiome of marine nematodes.** Metadata includes the habitat that the worms were isolated from, the genus-level identification of the worm, the genome amplification protocol, and the date they were collected from.

**Table S3: Accessory genes more commonly found in host-associated genomes.** A summary of the genes more commonly found in host-associated (t+) pigmented (orange) and nonpigmented (purple) genomes**.** The gene family ID is in bold, followed by a brief description of the product. The presence and absence of a function or gene is indicated by (g+) or (g-), respectively. Functional predictions of hypothetical proteins by Gaia, an AI agent developed by Tatta Bio that integrates several genomic and structural features to predict functions of hypothetical proteins, are in red. An odds-ratio greater than one indicates that the gene/function is more commonly associated with the host-associated lifestyle strains**.**

**Table S4: Metabolic functions and biogeochemical traits more commonly found in host-associated genomes.** A summary of the metabolic functions and enzymes associated with host-associated (t+) pigmented (orange) and nonpigmented (purple) genomes. The name of the enzyme or function is in bold, followed by a brief description of its function. The presence and absence of a function or gene is indicated by (g+) or (g-), respectively. An odds-ratio greater than one indicates that the gene/function is more commonly associated with the host-associated lifestyle strains.
