# Supplementary material for: Pseudoalteromonas is a symbiont of marine invertebrates that exhibits broad patterns of phylosymbiosis
Source: ISME J. 2026 Apr 20;20(1):wrag091. doi: 10.1093/ismejo/wrag091 (PMC13245728; doi:10.1093/ismejo/wrag091)
Supplement: Supplementary_material_wrag091 [file supplementary_material_wrag091.zip › Supplementary Table 1.docx]

**Supplementary Table 1: Summary of the genomes included in this study.** The lifestyle of the isolated strain (host-associated vs. free-living), the phyla of the host it was isolated from, the completeness (%) and contamination (%) according to CheckM, AccessionID, and citation where the genome was sequenced. Genomes without a citation was left as N/A. The two genomes isolated from marine nematodes in this study are highlighted in yellow.

| **SampleID** | **AccessionID** | **Phylogroup** | **Comp. (%)** | **Cont. (%)** | **Lifestyle** | **Host Phylum** | **Citation** |
| --- | --- | --- | --- | --- | --- | --- | --- |
| **P_1131a** | GCF_001401875.1 | PG_44 | 100 | 0.57 | Host-associated | Cnidaria | (Klassen, Wolf, et al., 2015) |
| **P_1161b** | GCF_001401785.1 | PG_35 | 99.95 | 0.25 | Host-associated | Cnidaria | (Klassen, Wolf, et al., 2015) |
| **P_125** | GCF_001401805.1 | PG_44 | 99.75 | 0.82 | Host-associated | Cnidaria | (Klassen, Wolf, et al., 2015) |
| **P_126** | GCF_001401795.1 | PG_30 | 100 | 0.73 | Host-associated | Cnidaria | (Klassen, Wolf, et al., 2015) |
| **P_130** | GCF_001400005.1 | PG_45 | 100 | 0.38 | Host-associated | Cnidaria | (Klassen, Rischer, et al., 2015) |
| **P_17a** | GCF_001401855.1 | PG_44 | 100 | 0.51 | Host-associated | Cnidaria | (Klassen, Wolf, et al., 2015) |
| **P_1CM17D** | GCF_023161805.1 | PG_45 | 100 | 0.25 | Host-associated | Cnidaria | PROBIOMA |
| **P_ACER1** | GCF_024124445.1 | PG_29 | 99.61 | 1.22 | Host-associated | Cnidaria | (Ushijima et al., 2023) |
| **P_APAL1** | GCF_021532475.1 | PG_26 | 99.70 | 1.07 | Host-associated | Cnidaria | (Meyer JL, 2024) |
| **P_2CM32C** | GCF_023161925.1 | PG_43 | 100 | 0.13 | Host-associated | Porifera | PROBIOMA |
| **P_2CM36K** | GCF_023161865.1 | PG_34 | 99.95 | 0.27 | Host-associated | Chordata | PROBIOMA |
| **P_B95** | GCF_030125375.1 | PG_03 | 99.95 | 2.63 | Host-associated | Cnidaria | (E. M. Thomas et al., 2025) |
| **P_BZA4** | GCF_040104395.1 | PG_30 | 99.70 | 0.63 | Host-associated | Cnidaria | (Meyer JL, 2024) |
| **P_P94** | GCF_030135505.1 | PG_02 | 99.70 | 0.41 | Host-associated | Cnidaria | (E. M. Thomas et al., 2025) |
| **P_piratica_OCN003** | GCF_000788395.1 | PG_55 | 100 | 1.31 | Host-associated | Cnidaria | (Beurmann et al., 2015) |
| **P_A22** | GCF_018141345.1 | PG_15 | 99.12 | 0.23 | Host-associated | Arthropoda | N/A |
| **P_A25** | GCF_009176705.1 | PG_22 | 99.75 | 0.23 | Free-living | Seawater | (Kitaguchi et al., 2019) |
| **P_AC163** | GCF_000497935.1 | PG_40 | 95.75 | 1.3 | Host-associated | Porifera | (Bosi et al., 2017) |
| **P_R96** | GCF_030125445.1 | PG_06 | 99.96 | 0.76 | Host-associated | Cnidaria | (E. M. Thomas et al., 2025) |
| **P_agarivorans_DSM14585** | GCF_002310855.1 | PG_45 | 99.20 | 0.25 | Free-living | Seawater | (Romanenko et al., 2003) |
| **P_agarivorans_NW4327** | GCF_000508785.1 | PG_45 | 100 | 3.79 | Host-associated | Porifera | (Choudhury et al., 2014) |
| **P_agarivorans_S816** | GCF_000363985.1 | PG_45 | 100 | 0.51 | Free-living | Seawater | (Gram et al., 2010) |
| **P_aliena_EH1** | GCF_001999225.1 | PG_38 | 100 | 0.62 | Free-living | Seawater | N/A |
| **P_aliena_SW19** | GCF_014905615.1 | PG_38 | 100 | 0.25 | Free-living | Seawater | (Ivanova et al., 2004) |
| **P_amylolytica_JW1** | GCF_001854605.1 | PG_21 | 99.49 | 0.54 | Free-living | Seawater | (Wu et al., 2017) |
| **P_2CM37A** | GCF_023161885.1 | PG_45 | 100 | 0.25 | Host-associated | Cnidaria | PROBIOMA |
| **P_APM04** | GCF_022809815.1 | PG_43 | 99.75 | 0.13 | Free-living | Seawater | (Taguchi et al., 2022) |
| **P_arctica_A3712** | GCF_000238395.3 | PG_42 | 99.75 | 0.38 | Free-living | Seawater | (B-B Xie et al., 2012) |
| **P_arctica_NECBIFX0059** | GCF_012844465.1 | PG_41 | 100 | 0.13 | Host-associated | Mollusca | N/A |
| **P_arctica_NECBIFX2020001** | GCF_012933495.1 | PG_42 | 100 | 0.13 | Host-associated | Mollusca | N/A |
| **P_arctica_NECBIFX2020017** | GCF_012935355.1 | PG_42 | 100 | 0.13 | Host-associated | Echinodermata | N/A |
| **P_aurantia_208** | GCF_014858725.1 | PG_20 | 100 | 0.48 | Free-living | Seawater | (Gauthier et al., 1995) |
| **P_aurantia_S3788** | GCF_005886875.1 | PG_18 | 99.92 | 0.44 | Free-living | Seawater | (Gram et al., 2010) |
| **P_aurantia_S3790** | GCF_005887355.1 | PG_18 | 99.66 | 0.69 | Free-living | Seawater | (Gram et al., 2010) |
| **P_aurantia_S3895** | GCF_005887285.1 | PG_18 | 99.92 | 0.51 | Free-living | Seawater | (Gram et al., 2010) |
| **P_2CM41L** | GCF_023161905.1 | PG_45 | 100 | 0.25 | Host-associated | Cnidaria | PROBIOMA |
| **P_flavipulchra_SCSIO43202** | GCF_024746855.1 | PG_15 | 100 | 0.41 | Host-associated | Cnidaria | (W. Wang et al., 2022) |
| **P_SCSIO43088** | GCF_023650795.1 | PG_30 | 98.05 | 0.32 | Host-associated | Cnidaria | (K. Tang et al., 2020) |
| **P_SCSIO43101** | GCF_023650775.1 | PG_27 | 99.95 | 0.46 | Host-associated | Cnidaria | (K. Tang et al., 2020) |
| **P_SCSIO43201** | GCF_023716725.1 | PG_13 | 99.75 | 1.22 | Host-associated | Cnidaria | (K. Tang et al., 2020) |
| **P_BMB** | GCF_001709235.1 | PG_15 | 99.47 | 0.34 | Host-associated | Ctenophora | N/A |
| **P_Cn537** | GCF_021532495.1 | PG_29 | 99.95 | 0.76 | Host-associated | Cnidaria | (Meyer JL, 2024) |
| **P_CNAT218** | GCF_021530715.1 | PG_16 | 99.37 | 0.97 | Host-associated | Cnidaria | (Deutsch et al., 2022) |
| **P_CNAT2181** | GCF_022347865.1 | PG_16 | 99.37 | 0.97 | Host-associated | Cnidaria | (Deutsch et al., 2022) |
| **P_C2R02** | GCF_018860845.1 | PG_57 | 98.70 | 3.70 | Free-living | Seawater | N/A |
| **P_citrea_DSM8771** | GCF_000238375.3 | PG_19 | 100 | 0.25 | Free-living | Seawater | N/A |
| **P_citrea_S2231** | GCF_005887475.1 | PG_17 | 99.92 | 0.65 | Host-associated | Arthropoda | (Paulsen et al., 2019) |
| **P_citrea_S2233** | GCF_005887445.1 | PG_17 | 99.92 | 0.79 | Host-associated | Arthropoda | (Paulsen et al., 2019) |
| **P_CNAT241** | GCF_040104515.1 | PG_16 | 99.37 | 0.97 | Host-associated | Cnidaria | (Deutsch et al., 2022) |
| **P_bablabjr011** | GCF_016464045.1 | PG_24 | 99.95 | 0.51 | Host-associated | Cnidaria | (Babbin et al., 2021) |
| **P_DL2H1** | GCF_021532565.1 | PG_15 | 99.49 | 0.39 | Host-associated | Cnidaria | (Deutsch et al., 2022) |
| **P_DL2H22** | GCF_021532535.1 | PG_09 | 99.96 | 0.25 | Host-associated | Cnidaria | (Deutsch et al., 2022) |
| **P_DL2H6** | GCF_021530725.1 | PG_15 | 99.13 | 0.39 | Host-associated | Cnidaria | (Deutsch et al., 2022) |
| **P_shioyasakiensis_28** | GCF_024105465.1 | PG_30 | 97.50 | 0.34 | Host-associated | Cnidaria | (Varasteh et al., 2021) |
| **P_BZB3** | GCF_040104455.1 | PG_50 | 98.69 | 0.51 | Host-associated | Cnidaria | (Meyer JL, 2024) |
| **P_Ps84H4** | GCF_024124645.1 | PG_26 | 99.70 | 1.25 | Host-associated | Cnidaria | (Meyer JL, 2024) |
| **P_CO109Y** | GCF_004208935.1 | PG_30 | 99.95 | 0.53 | Host-associated | Cnidaria | (Atencio et al., 2020) |
| **P_CO133X** | GCF_004208945.1 | PG_25 | 99.95 | 0.13 | Host-associated | Cnidaria | (Atencio et al., 2020) |
| **P_CO302Y** | GCF_004209095.1 | PG_25 | 99.95 | 0.13 | Host-associated | Cnidaria | (Atencio et al., 2020) |
| **P_CO325X** | GCF_004208955.1 | PG_16 | 99.49 | 0.72 | Host-associated | Cnidaria | (Atencio et al., 2020) |
| **P_CO342X** | GCF_004208915.1 | PG_15 | 100 | 0.16 | Host-associated | Cnidaria | (Atencio et al., 2020) |
| **P_CO348** | GCF_004208845.1 | PG_15 | 100 | 0.70 | Host-associated | Cnidaria | (Atencio et al., 2020) |
| **P_D15MCD2** | GCF_038562745.1 | PG_30 | 99.62 | 0.77 | Free-living | Seawater | N/A |
| **P_distincta_16SW7** | GCF_005877035.1 | PG_40 | 100 | 0.38 | Free-living | Seawater | (Nedashkovkaya et al., 2021) |
| **P_distincta_22A13** | GCF_028414575.1 | PG_40 | 100 | 0.13 | Host-associated | Echinodermata | N/A |
| **P_distincta_ATCC700518** | GCF_000814675.1 | PG_40 | 99.75 | 0.13 | Host-associated | Porifera | (Givan et al., 2015) |
| **P_distincta_KMM3548** | GCF_014918315.1 | PG_40 | 98.70 | 0.13 | Host-associated | Cnidaria | (Ivanova, Sawabe, Lysenko, Gorshkova, Hayashi, et al., 2002) |
| **P_distincta_LMG14908** | GCF_030710225.1 | PG_40 | 100 | 0.13 | Host-associated | Mollusca | (Videau et al., 2024) |
| **P_B7P1** | GCF_040104415.1 | PG_30 | 99.70 | 0.98 | Host-associated | Cnidaria | (Meyer JL, 2024) |
| **P_CNC920** | GCF_022347875.1 | PG_16 | 99.37 | 0.72 | Host-associated | Cnidaria | (Meyer JL, 2024) |
| **P_MM172** | GCF_022347945.1 | PG_16 | 99.37 | 0.97 | Host-associated | Cnidaria | (Meyer JL, 2024) |
| **P_donghaensis_HJ51** | GCF_003515105.1 | PG_24 | 99.95 | 1.01 | Free-living | Seawater | N/A |
| **P_DSM17587** | GCF_000238335.3 | PG_43 | 100 | 0.13 | Free-living | Seawater | (Nam et al., 2007) |
| **P_EB27** | GCF_001974875.1 | PG_41 | 99.75 | 0.13 | Host-associated | Porifera | (Borchert et al., 2017) |
| **P_ECSMB14103** | GCF_000813065.1 | PG_43 | 100 | 0.13 | Host-associated | Mollusca | (Guo et al., 2015) |
| **P_EKP108** | GCF_014534795.1 | PG_40 | 100 | 0.38 | Free-living | Seawater | (Phillips et al., 2023) |
| **P_espejiana_DSM9414** | GCF_002221525.1 | PG_46 | 99.45 | 0.51 | Free-living | Seawater | (Videau et al., 2024) |
| **P_FE4** | GCF_030297135.1 | PG_27 | 99.95 | 0.13 | Host-associated | Echinodermata | (Kudo et al., 2024) |
| **P_flavipulchra_ATCCBAA314** | GCF_000814665.1 | PG_15 | 99.75 | 1.30 | Free-living | Seawater | (Givan et al., 2015) |
| **P_flavipulchra_LMG20361** | GCF_014596995.1 | PG_15 | 95.08 | 0.32 | Free-living | Seawater | (Paulsen et al., 2020) |
| **P_flavipulchra_NCIMB2033** | GCF_014858715.1 | PG_15 | 99.75 | 0.90 | Free-living | Seawater | N/A |
| **P_flavipulchra_S1925** | GCF_005886245.1 | PG_15 | 100 | 1.17 | Host-associated | Arthropoda | (Machado et al., 2015) |
| **P_BZK2** | GCF_014349195.1 | PG_29 | 99.99 | 0.61 | Host-associated | Cnidaria | (Sweet et al., 2021) |
| **P_fuliginea_KMM216** | GCF_000690055.1 | PG_39 | 99.75 | 0.57 | Host-associated | Chordata | (Machado et al., 2015) |
| **P_fuliginea_S2292** | GCF_000967705.1 | PG_39 | 99.75 | 0.57 | Host-associated | Porifera | (Machado et al., 2015) |
| **P_G4** | GCF_028875915.1 | PG_54 | 98.57 | 1.54 | Host-associated | Arthropoda | (L. Chen et al., 2025) |
| **P_galatheae_S4498** | GCF_005886105.2 | PG_14 | 99.75 | 0.67 | Host-associated | Arthropoda | (Paulsen et al., 2020) |
| **P_gelatinilytica_NH153** | GCF_001641615.1 | PG_28 | 99.95 | 0.65 | Free-living | Seawater | (Yan et al., 2016) |
| **P_Hal040** | GCF_037162425.1 | PG_28 | 95.03 | 0.41 | Host-associated | Porifera | (Galarza-Verkovitch et al., 2023) |
| **P_HMSA03** | GCF_002289345.1 | PG_15 | 97.55 | 0.19 | Host-associated | Mollusca | (Chau et al., 2021) |
| **P_JC28** | GCF_013319255.1 | PG_15 | 99.49 | 0.38 | Host-associated | Mollusca | (Suria et al., 2020) |
| **P_JC3** | GCF_017168135.1 | PG_15 | 99.75 | 1.36 | Host-associated | Arthropoda | (Rosario et al., 2021) |
| **P_JC31** | GCF_030291895.1 | PG_15 | 99.75 | 1.36 | Host-associated | Arthropoda | N/A |
| **P_JW3** | GCF_001854555.1 | PG_21 | 99.49 | 0.54 | Free-living | Seawater | (Wu et al., 2017) |
| **P_kknpp56** | GCF_018598885.1 | PG_34 | 99.77 | 0.76 | Free-living | Seawater | (Rajeev et al., 2021) |
| **P_KMM661** | GCF_002221505.1 | PG_37 | 99.89 | 0.54 | Host-associated | Mollusca | N/A |
| **P_lipolytica_CSB02KR** | GCF_001704875.1 | PG_24 | 99.95 | 0.48 | Host-associated | Echinodermata | (Jo et al., 2017) |
| **P_lipolytica_LMEB39** | GCF_014925285.1 | PG_24 | 99.87 | 0.23 | Free-living | Seawater | N/A |
| **P_luteoviolacea_2ta16** | GCF_000495575.1 | PG_01 | 98.94 | 1.70 | Host-associated | Cnidaria | N/A |
| **P_M1318928** | GCF_022398505.1 | PG_27 | 99.70 | 0.41 | Host-associated | Mollusca | (Worden et al., 2022) |
| **P_M1400202** | GCF_022398435.1 | PG_14 | 99.46 | 0.9 | Host-associated | Mollusca | (Worden et al., 2022) |
| **P_M8** | GCF_018141365.1 | PG_15 | 97.85 | 0.98 | Host-associated | Arthropoda | N/A |
| **P_maricaloris_LMG19692** | GCF_012641725.1 | PG_15 | 99.66 | 0.21 | Host-associated | Porifera | (Paulsen et al., 2020) |
| **P_MB47** | GCF_011319765.1 | PG_29 | 99.95 | 0.25 | Host-associated | Echinodermata | (Mikkelsen et al., 2021) |
| **P_OANN1** | GCF_024124685.1 | PG_15 | 99.75 | 0.18 | Host-associated | Cnidaria | (Meyer JL, 2024) |
| **P_Of11M6** | GCF_022347905.1 | PG_15 | 99.73 | 0.73 | Host-associated | Cnidaria | (Meyer JL, 2024) |
| **P_MEBiC03607** | GCF_004792295.1 | PG_26 | 99.95 | 0.72 | Free-living | Seawater | N/A |
| **P_OF5H5** | GCF_021530695.1 | PG_15 | 99.75 | 0.39 | Host-associated | Cnidaria | (Meyer JL, 2024) |
| **P_MMG024** | GCF_021654175.1 | PG_53 | 99.75 | 0.63 | Host-associated | Annelida | (Alker et al., 2021) |
| **P_N12309** | GCF_032716425.1 | PG_25 | 99.95 | 0.18 | Free-living | Seawater | N/A |
| **P_NBRC102222** | GCF_007989585.1 | PG_46 | 99.49 | 0.51 | Free-living | Seawater | N/A |
| **P_NBRC12985** | GCF_006539245.1 | PG_47 | 99.84 | 0.25 | Free-living | Seawater | N/A |
| **P_neustronica_PAMC28425** | GCF_001653135.1 | PG_33 | 99.75 | 0.88 | Free-living | Seawater | (Hwang et al., 2016) |
| **P_neustronica_SM1927** | GCF_007786355.1 | PG_32 | 99.75 | 1.03 | Free-living | Seawater | N/A |
| **P_NJ631** | GCF_000276645.1 | PG_15 | 100 | 0.16 | Host-associated | Porifera | (Chen W. et al., 2012) |
| **P_Of5H6** | GCF_040104565.1 | PG_15 | 99.75 | 0.39 | Host-associated | Cnidaria | (Deutsch et al., 2022) |
| **P_OF7H1** | GCF_022347825.1 | PG_15 | 100 | 0.53 | Host-associated | Cnidaria | (Deutsch et al., 2022) |
| **P_Of7M16** | GCF_022347805.1 | PG_01 | 99.70 | 0.51 | Host-associated | Cnidaria | (Meyer JL, 2024) |
| **P_OFAV1** | GCF_021532595.1 | PG_26 | 99.66 | 3.78 | Host-associated | Cnidaria | (Meyer JL, 2024) |
| **P_OOF1S7** | GCF_022347765.1 | PG_11 | 99.71 | 0.49 | Host-associated | Cnidaria | (Meyer JL, 2024) |
| **P_bablabjr004** | GCF_016464215.1 | PG_26 | 99.70 | 0.74 | Host-associated | Cnidaria | (Babbin et al., 2021) |
| **P_CnMc713** | GCF_040104495.1 | PG_16 | 99.37 | 0.72 | Host-associated | Cnidaria | (Meyer JL, 2024) |
| **P_CnMc715** | GCF_022347925.1 | PG_16 | 99.37 | 0.72 | Host-associated | Cnidaria | (Meyer JL, 2024) |
| **P_ostereae_hOe124** | GCF_029023665.1 | PG_31 | 99.12 | 1.26 | Host-associated | Mollusca | (Cuny et al., 2021) |
| **P_ostereae_hOe125** | GCF_029026505.1 | PG_31 | 99.12 | 1.26 | Host-associated | Mollusca | (Cuny et al., 2021) |
| **P_ostereae_hOe66** | GCF_018069805.1 | PG_31 | 99.12 | 1.26 | Host-associated | Mollusca | (Cuny et al., 2021) |
| **P_P111** | GCF_001399995.1 | PG_45 | 100 | 0.81 | Host-associated | Cnidaria | (Klassen, Rischer, et al., 2015) |
| **P_P18** | GCF_001399985.1 | PG_30 | 100 | 0.74 | Host-associated | Cnidaria | (Klassen, Rischer, et al., 2015) |
| **P_P19** | GCF_001399975.1 | PG_53 | 99.75 | 0.82 | Host-associated | Cnidaria | (Klassen, Wolf, et al., 2015) |
| **P_CnMc737** | GCF_024124485.1 | PG_30 | 99.95 | 1.01 | Host-associated | Cnidaria | (Meyer JL, 2024) |
| **P_PA2MD11** | GCF_017808125.1 | PG_29 | 99.95 | 0.25 | Host-associated | Porifera | (de Oliveira et al., 2021) |
| **P_McH142** | GCF_022347845.1 | PG_05 | 99.96 | 0.51 | Host-associated | Cnidaria | (Meyer JL, 2024) |
| **P_peptidolytica_DSM14001** | GCF_012641745.1 | PG_13 | 95.32 | 1.22 | Free-living | Seawater | (Paulsen et al., 2020) |
| **P_peptidolytica_F1250A1** | GCF_014858745.1 | PG_13 | 99.49 | 1.22 | Free-living | Seawater | N/A |
| **P_peptidolytica_NBRC101021** | GCF_007989895.1 | PG_13 | 99.49 | 1.22 | Free-living | Seawater | N/A |
| **P_phenolica_KCTC12086** | GCF_001444405.1 | PG_49 | 99.95 | 1.12 | Free-living | Seawater | (Choe et al., 2016) |
| **P_phenolica_OBC302** | GCF_014925335.1 | PG_49 | 99.95 | 1.12 | Free-living | Seawater | N/A |
| **P_phenolica_S1093** | GCF_005886345.1 | PG_48 | 99.95 | 0.84 | Free-living | Seawater | (Paulsen et al., 2019) |
| **P_phenolica_S1189** | GCF_005887275.1 | PG_48 | 99.95 | 2.24 | Free-living | Seawater | (Paulsen et al., 2019) |
| **P_phenolica_S3663** | GCF_005876835.1 | PG_51 | 99.95 | 0.57 | Free-living | Seawater | (Paulsen et al., 2019) |
| **P_phenolica_S3898** | GCF_004214845.1 | PG_48 | 99.70 | 1.02 | Free-living | Seawater | (Paulsen et al., 2019) |
| **P_McH17** | GCF_013366255.1 | PG_13 | 99.75 | 1.47 | Host-associated | Cnidaria | (Ushijima et al., 2023) |
| **P_porphyrae_MNAD16** | GCF_003591235.1 | PG_28 | 99.95 | 0.53 | Free-living | Seawater | N/A |
| **P_PPB1** | GCF_015356275.1 | PG_05 | 99.01 | 0.25 | Host-associated | Porifera | (Sakai-Kawada et al., 2020) |
| **P_SMS1** | GCF_021530565.1 | PG_04 | 99.95 | 1.28 | Host-associated | Cnidaria | (Meyer JL, 2024) |
| **P_XMcav11Q** | GCF_040104525.1 | PG_15 | 99.75 | 1.14 | Host-associated | Cnidaria | (Meyer JL, 2024) |
| **P_rhizosphaerae_hCg42** | GCF_028885455.1 | PG_32 | 100 | 4.86 | Host-associated | Mollusca | N/A |
| **P_rubra_DSM6842** | GCF_000238295.3 | PG_07 | 99.71 | 0.99 | Free-living | Seawater | (Bin-Bin Xie et al., 2012) |
| **P_rubra_S1946** | GCF_004212645.1 | PG_10 | 99.71 | 0.18 | Host-associated | Arthropoda | (Paulsen et al., 2019) |
| **P_rubra_S2471** | GCF_000967655.1 | PG_09 | 99.96 | 0.91 | Host-associated | Mollusca | (Machado et al., 2015) |
| **P_rubra_SCSIO_6842** | GCF_001482385.1 | PG_06 | 99.96 | 0.34 | Free-living | Seawater | (Li et al., 2016) |
| **P_ruthenica_LMG19699** | GCF_008808095.1 | PG_16 | 98.85 | 1.47 | Host-associated | Mollusca | (Ivanova, Sawabe, Lysenko, Gorshkova, Svetashev, et al., 2002) |
| **P_ruthenica_S2756** | GCF_005876865.1 | PG_16 | 99.37 | 0.78 | Host-associated | Mollusca | (Paulsen et al., 2019) |
| **P_ruthenica_S3245** | GCF_005886975.1 | PG_16 | 99.49 | 2.52 | Host-associated | Arthropoda | (Paulsen et al., 2019) |
| **P_S2893** | GCF_005887105.1 | PG_45 | 100 | 0.79 | Host-associated | Echinodermata | (Paulsen et al., 2019) |
| **P_S3173** | GCF_005930795.1 | PG_34 | 99.67 | 0.76 | Host-associated | Cnidaria | (Paulsen et al., 2019) |
| **P_S3178** | GCF_005886985.1 | PG_47 | 99.96 | 2.25 | Host-associated | Arthropoda | (Paulsen et al., 2019) |
| **P_S3260** | GCF_005886925.1 | PG_34 | 99.95 | 1.49 | Host-associated | Arthropoda | (Paulsen et al., 2019) |
| **P_Scap03** | GCF_013393115.1 | PG_35 | 97.85 | 0.84 | Host-associated | Porifera | (Mead et al., 2021) |
| **P_scap25** | GCF_013394125.1 | PG_35 | 99.95 | 0.84 | Host-associated | Porifera | (Mead et al., 2021) |
| **P_scap26** | GCF_013394165.1 | PG_35 | 99.95 | 0.84 | Host-associated | Porifera | (Mead et al., 2021) |
| **P_SCSIO11900** | GCF_000576475.1 | PG_34 | 99.95 | 0.38 | Host-associated | Cnidaria | (Zeng et al., 2014) |
| **P_XMcav2N** | GCF_024124545.1 | PG_08 | 99.92 | 0.38 | Host-associated | Cnidaria | (Meyer JL, 2024) |
| **P_XMcav2N2** | GCF_040104535.1 | PG_08 | 99.92 | 0.38 | Host-associated | Cnidaria | (Meyer JL, 2024) |
| **P_2102** | GCF_013350045.1 | PG_30 | 99.95 | 0.44 | Host-associated | Cnidaria | (Sweet et al., 2021) |
| **P_2103** | GCF_013349985.1 | PG_30 | 99.95 | 0.44 | Host-associated | Cnidaria | (Sweet et al., 2021) |
| **P_SDCH90** | GCF_019134595.1 | PG_30 | 99.44 | 0.99 | Host-associated | Mollusca | N/A |
| **P_SG411** | GCF_014164575.1 | PG_32 | 99.75 | 1.05 | Free-living | Seawater | N/A |
| **P_SG412** | GCF_014164535.1 | PG_32 | 99.87 | 1.03 | Free-living | Seawater | N/A |
| **P_SG415** | GCF_014164495.1 | PG_32 | 98.97 | 1.72 | Free-living | Seawater | N/A |
| **P_SG416** | GCF_014164455.1 | PG_38 | 99.73 | 0.41 | Free-living | Seawater | N/A |
| **P_SG418** | GCF_014164425.1 | PG_32 | 99.62 | 1.53 | Free-living | Seawater | N/A |
| **P_SG431** | GCF_014164415.1 | PG_40 | 99.94 | 0.37 | Free-living | Seawater | N/A |
| **P_SG433** | GCF_014164385.1 | PG_40 | 100 | 0.21 | Free-living | Seawater | N/A |
| **P_SG434** | GCF_014164325.1 | PG_40 | 99.75 | 0.22 | Free-living | Seawater | N/A |
| **P_SG435** | GCF_014164345.1 | PG_40 | 100 | 0.13 | Free-living | Seawater | N/A |
| **P_SG436** | GCF_014164365.1 | PG_32 | 99.87 | 1.56 | Free-living | Seawater | N/A |
| **P_SG437** | GCF_014164295.1 | PG_32 | 99.37 | 1.31 | Free-living | Seawater | N/A |
| **P_SG438** | GCF_014164245.1 | PG_40 | 99.92 | 0.16 | Free-living | Seawater | N/A |
| **P_SG441** | GCF_014164315.1 | PG_32 | 99.87 | 1.65 | Free-living | Seawater | N/A |
| **P_SG4417** | GCF_014164235.1 | PG_32 | 99.96 | 0.86 | Free-living | Seawater | N/A |
| **P_SG444** | GCF_014164225.1 | PG_38 | 100 | 0.38 | Free-living | Seawater | N/A |
| **P_SG445** | GCF_014164175.1 | PG_37 | 99.97 | 0.32 | Free-living | Seawater | N/A |
| **P_SG448** | GCF_014164125.1 | PG_32 | 100 | 0.74 | Free-living | Seawater | N/A |
| **P_SG451** | GCF_014164135.1 | PG_40 | 100 | 0.41 | Free-living | Seawater | N/A |
| **P_SG452** | GCF_014164115.1 | PG_40 | 100 | 0.13 | Free-living | Seawater | N/A |
| **P_SG453** | GCF_014164075.1 | PG_40 | 100 | 0.21 | Free-living | Seawater | N/A |
| **P_SG455** | GCF_014164085.1 | PG_38 | 100 | 0.13 | Free-living | Seawater | N/A |
| **P_SG456** | GCF_014164025.1 | PG_40 | 99.66 | 0.71 | Free-living | Seawater | N/A |
| **P_0303** | GCF_013349995.1 | PG_30 | 99.95 | 0.72 | Host-associated | Cnidaria | (Sweet et al., 2021) |
| **P_0802** | GCF_013350085.1 | PG_30 | 99.95 | 0.44 | Host-associated | Cnidaria | (Sweet et al., 2021) |
| **P_shioyasakiensis_BMC2** | GCF_029992275.1 | PG_30 | 99.95 | 0.44 | Host-associated | Cnidaria | (Rosado et al., 2023) |
| **P_shioyasakiensis_BMC3** | GCF_029992495.1 | PG_30 | 99.89 | 0.44 | Host-associated | Cnidaria | (Rosado et al., 2023) |
| **P_shioyasakiensis_BMC4** | GCF_029991435.1 | PG_30 | 99.95 | 0.80 | Host-associated | Cnidaria | (Rosado et al., 2023) |
| **P_shioyasakiensis_G21653S1** | GCF_027213805.1 | PG_30 | 99.95 | 0.79 | Host-associated | Cnidaria | (Domin et al., 2023) |
| **P_shioyasakiensis_LC2** | GCF_030247135.1 | PG_30 | 99.95 | 1.36 | Free-living | Seawater | (Villela et al., 2023) |
| **P_shioyasakiensis_M1400201** | GCF_022398475.1 | PG_30 | 99.49 | 0.68 | Host-associated | Mollusca | N/A |
| **P_SiA1** | GCF_018732205.1 | PG_34 | 99.44 | 0.51 | Host-associated | Echinodermata | N/A |
| **P_SK18** | GCF_001974855.1 | PG_36 | 99.70 | 0.51 | Host-associated | Porifera | (Borchert et al., 2017) |
| **P_SK20** | GCF_001974845.1 | PG_34 | 99.92 | 0.74 | Host-associated | Porifera | (Borchert et al., 2017) |
| **P_shioyasakiensis_BMC5** | GCF_029991425.1 | PG_30 | 99.95 | 0.44 | Host-associated | Cnidaria | (Rosado et al., 2023) |
| **P_spongiae_SAO44** | GCF_002814155.1 | PG_52 | 100 | 2.17 | Free-living | Seawater | (B.-L. Tang et al., 2018) |
| **P_spongiae_UST010723006** | GCF_000238255.3 | PG_52 | 100 | 0.38 | Host-associated | Porifera | (B-B Xie et al., 2012) |
| **P_SR411** | GCF_014164015.1 | PG_40 | 100 | 0.16 | Free-living | Seawater | N/A |
| **P_SR414** | GCF_014163985.1 | PG_32 | 99.75 | 0.83 | Free-living | Seawater | N/A |
| **P_SR415** | GCF_014163975.1 | PG_40 | 100 | 0.16 | Free-living | Seawater | N/A |
| **P_SR416** | GCF_014163945.1 | PG_32 | 99.62 | 1.56 | Free-living | Seawater | N/A |
| **P_SR417** | GCF_014163935.1 | PG_40 | 100 | 0.24 | Free-living | Seawater | N/A |
| **P_SR418** | GCF_014163915.1 | PG_32 | 100 | 0.92 | Free-living | Seawater | N/A |
| **P_SR432** | GCF_014163885.1 | PG_40 | 100 | 0.21 | Free-living | Seawater | N/A |
| **P_SR433** | GCF_014163875.1 | PG_40 | 99.24 | 0.13 | Free-living | Seawater | N/A |
| **P_SR435** | GCF_014163835.1 | PG_40 | 100 | 0.16 | Free-living | Seawater | N/A |
| **P_SR436** | GCF_014163815.1 | PG_40 | 100 | 0.16 | Free-living | Seawater | N/A |
| **P_SR437** | GCF_014163785.1 | PG_40 | 100 | 0.57 | Free-living | Seawater | N/A |
| **P_SR442** | GCF_014163745.1 | PG_40 | 99.87 | 0.16 | Free-living | Seawater | N/A |
| **P_SR445** | GCF_014163675.1 | PG_32 | 99.62 | 1.56 | Free-living | Seawater | N/A |
| **P_SR448** | GCF_014163635.1 | PG_32 | 100 | 0.96 | Free-living | Seawater | N/A |
| **P_SR451** | GCF_014163655.1 | PG_40 | 100 | 0.47 | Free-living | Seawater | N/A |
| **P_SR454** | GCF_014163535.1 | PG_37 | 99.72 | 1.06 | Free-living | Seawater | N/A |
| **P_SR455** | GCF_014163565.1 | PG_40 | 96.96 | 1.61 | Free-living | Seawater | N/A |
| **P_SR456** | GCF_014163545.1 | PG_32 | 99.62 | 1.65 | Free-living | Seawater | N/A |
| **P_SW010604** | GCF_001293805.1 | PG_16 | 99.12 | 1.03 | Free-living | Seawater | N/A |
| **P_TB25** | GCF_000497995.1 | PG_40 | 95.32 | 0.73 | Host-associated | Porifera | (Bosi et al., 2017) |
| **P_tetradonis_CSB01KR** | GCF_001723425.1 | PG_34 | 99.95 | 0.13 | Host-associated | Echinodermata | (Jo et al., 2017) |
| **P_translucida_KMM520** | GCF_001465295.1 | PG_37 | 100 | 0.04 | Free-living | Seawater | (Rong et al., 2016) |
| **P_tunicata_D2** | GCF_002310815.1 | PG_56 | 99.75 | 01.3 | Host-associated | Chordata | (T. Thomas et al., 2008) |
| **P_UG31** | GCF_037120685.1 | PG_12 | 99.66 | 0.85 | Host-associated | Arthropoda | (Yang et al., 2024) |
| **P_UG32** | GCF_037120705.1 | PG_12 | 100 | 0.18 | Host-associated | Arthropoda | (Yang et al., 2024) |
| **P_undina_DSM6065** | GCF_000238275.3 | PG_35 | 99.87 | 0.25 | Free-living | Seawater | (B-B Xie et al., 2012) |
| **P_undina_NEM01** | GCF_048401115.1 | PG_35 | 100 | 0.71 | Host-associated | Nematoda | (De Santiago et al., 2025) |
| **P_undina_NEM02** | GCF_048401065.1 | PG_35 | 100 | 0.71 | Host-associated | Nematoda | (De Santiago et al., 2025) |
| **P_viridis_BBR56** | GCF_017742995.1 | PG_10 | 99.20 | 0.51 | Free-living | Seawater | (Handayani et al., 2024) |
| **P_xiamenensis_PSDB** | GCF_030994125.1 | PG_23 | 98.08 | 2.31 | Host-associated | Mollusca | N/A |
| **P_bablabjr010** | GCF_016464105.1 | PG_29 | 99.95 | 0.75 | Host-associated | Cnidaria | (Babbin et al., 2021) |
| **P_PAST1** | GCF_021532515.1 | PG_29 | 99.49 | 1.01 | Host-associated | Cnidaria | (Meyer JL, 2024) |
| **P_SCSIO43095** | GCF_023650455.1 | PG_34 | 99.02 | 0.63 | Host-associated | Cnidaria | (P. Wang et al., 2017) |

Alker, A. T., Gode, B. S., Aspiras, A. E., Jones, J. E., Michael, S. R., Aguilar, D., Cain, A. D., Candib, A. M., Cizmic, J. M., Clark, E. A., Cozzo, A. C., Figueroa, L. E., Garcia, P. A., Heaney, C. M., Levy, A. T., Macknight, L., McCarthy, A. S., McNamara, J. P., Nguyen, K. A., … Shikuma, N. J. (2021). Draft genome sequences of 10 bacteria from the marine Pseudoalteromonas group. *Microbiology Resource Announcements*, *10*(32), e0040421.

Atencio, L. A., Boya P, C. A., Martin H, C., Mejía, L. C., Dorrestein, P. C., & Gutiérrez, M. (2020). Genome mining, microbial interactions, and molecular networking reveals new dibromoalterochromides from strains of Pseudoalteromonas of Coiba National Park-panama. *Marine Drugs*, *18*(9), 456.

Babbin, A. R., Tamasi, T., Dumit, D., Weber, L., Rodríguez, M. V. I., Schwartz, S. L., Armenteros, M., Wankel, S. D., & Apprill, A. (2021). Discovery and quantification of anaerobic nitrogen metabolisms among oxygenated tropical Cuban stony corals. *The ISME Journal*, *15*(4), 1222–1235.

Beurmann, S., Videau, P., Ushijima, B., Smith, A. M., Aeby, G. S., Callahan, S. M., & Belcaid, M. (2015). Complete genome sequence of Pseudoalteromonas sp. Strain OCN003, isolated from Kāne’ohe Bay, O’ahu, Hawaii. *Genome Announcements*, *3*(1). https://doi.org/10.1128/genomeA.01396-14

Borchert, E., Knobloch, S., Dwyer, E., Flynn, S., Jackson, S. A., Jóhannsson, R., Marteinsson, V. T., O’Gara, F., & Dobson, A. D. W. (2017). Biotechnological potential of cold adapted Pseudoalteromonas spp. Isolated from “deep sea” sponges. *Marine Drugs*, *15*(6), 184.

Bosi, E., Fondi, M., Orlandini, V., Perrin, E., Maida, I., de Pascale, D., Tutino, M. L., Parrilli, E., Lo Giudice, A., Filloux, A., & Fani, R. (2017). The pangenome of (Antarctic) Pseudoalteromonas bacteria: evolutionary and functional insights. *BMC Genomics*, *18*(1), 93.

Chau, R., Pearson, L. A., Cain, J., Kalaitzis, J. A., & Neilan, B. A. (2021). A Pseudoalteromonas clade with remarkable biosynthetic potential. *Applied and Environmental Microbiology*, *87*(6). https://doi.org/10.1128/AEM.02604-20

Chen, L., Huang, Z., Middelboe, M., Deng, D., & Ma, Y. (2025). Synergistic effects of commensals and phage predation in suppressing colonization by pathogenic Vibrio parahaemolyticus. *Npj Biofilms and Microbiomes*, *11*(1), 163.

Chen W., Zhu P., He S., Jin H., & Yan X. (2012). Nonribosomal peptides synthetases gene clusters and core domain in Pseudoalteromonas sp. NJ631. *Wei sheng wu xue bao [Acta microbiologica Sinica]*, *52*(12), 1531–1539.

Choe, H., Lee, S.-H., Kim, S.-G., Park, D.-S., Nasir, A., & Kim, K. M. (2016). Complete genome of Pseudoalteromonas phenolica KCTC 12086(T) (= O-BC30(T)), a marine bacterium producing polybrominated aromatic compounds. *Journal of Biotechnology*, *218*, 23–24.

Choudhury, J. D., Pramanik, A., Webster, N. S., Llewellyn, L. E., Gachhui, R., & Mukherjee, J. (2014). Draft genome sequence of Pseudoalteromonas sp. Strain NW 4327 (MTCC 11073, DSM 25418), a pathogen of the Great Barrier Reef sponge Rhopaloeides odorabile. *Genome Announcements*, *2*(1). https://doi.org/10.1128/genomeA.00001-14

Cuny, H., Offret, C., Boukerb, A. M., Parizadeh, L., Lesouhaitier, O., Le Chevalier, P., Jégou, C., Bazire, A., Brillet, B., & Fleury, Y. (2021). Pseudoalteromonas ostreae sp. nov., a new bacterial species harboured by the flat oyster Ostrea edulis. *International Journal of Systematic and Evolutionary Microbiology*, *71*(11). https://doi.org/10.1099/ijsem.0.005070

de Oliveira, B. F. R., Lopes, I. R., Canellas, A. L. B., Muricy, G., Jackson, S. A., Dobson, A. D. W., & Laport, M. S. (2021). Genomic and in silico protein structural analyses provide insights into marine polysaccharide-degrading enzymes in the sponge-derived Pseudoalteromonas sp. PA2MD11. *International Journal of Biological Macromolecules*, *191*, 973–995.

De Santiago, A., Barnes, S. J., Pereira, T. J., Marcelino-Barros, M., Bik, H. M., & Thrash, J. C. (2025). Complete genome sequences of two Pseudoalteromonas undina strains isolated from a marine nematode (Oncholaimidae) collected at Tybee Island. *Microbiology Resource Announcements*, *14*(8), e0041925.

Deutsch, J. M., Mandelare-Ruiz, P., Yang, Y., Foster, G., Routhu, A., Houk, J., De La Flor, Y. T., Ushijima, B., Meyer, J. L., Paul, V. J., & Garg, N. (2022). Metabolomics approaches to dereplicate natural products from coral-derived bioactive bacteria. *Journal of Natural Products*, *85*(3), 462–478.

Domin, H., Zimmermann, J., Taubenheim, J., Fuentes Reyes, G., Saueressig, L., Prasse, D., Höppner, M., Schmitz, R. A., Hentschel, U., Kaleta, C., & Fraune, S. (2023). Sequential host-bacteria and bacteria-bacteria interactions determine the microbiome establishment of Nematostella vectensis. *Microbiome*, *11*(1), 257.

Galarza-Verkovitch, D., Turak, O., Wiese, J., Rahn, T., Hentschel, U., & Borchert, E. (2023). Bioprospecting for polyesterase activity relevant for PET degradation in marine Enterobacterales isolates. *AIMS Microbiology*, *9*(3), 518–539.

Gauthier, G., Gauthier, M., & Christen, R. (1995). Phylogenetic analysis of the genera Alteromonas, Shewanella, and Moritella using genes coding for small-subunit rRNA sequences and division of the genus Alteromonas into two genera, Alteromonas (emended) and Pseudoalteromonas gen. nov., and proposal of twelve new species combinations. *International Journal of Systematic Bacteriology*, *45*(4), 755–761.

Givan, S. A., Zhou, M.-Y., Bromert, K., Bivens, N., & Chapman, L. F. (2015). Genome sequences of *Pseudoalteromonas* strains ATCC BAA-314, ATCC 70018, and ATCC 70019. *Genome Announcements*, *3*(3). https://doi.org/10.1128/genomea.00390-15

Gram, L., Melchiorsen, J., & Bruhn, J. B. (2010). Antibacterial activity of marine culturable bacteria collected from a global sampling of ocean surface waters and surface swabs of marine organisms. *Marine Biotechnology (New York, N.Y.)*, *12*(4), 439–451.

Guo, X.-P., Ding, D.-W., Bao, W.-Y., & Yang, J.-L. (2015). Draft genome sequence of Pseudoalteromonas sp. Strain ECSMB14103, isolated from the East China Sea. *Genome Announcements*, *3*(2). https://doi.org/10.1128/genomeA.00330-15

Handayani, D. P., Isnansetyo, A., & Istiqomah, I. (2024). New investigation of encoding secondary metabolites gene by genome mining of a marine bacterium, Pseudoalteromonas viridis BBR56. *BMC Genomics*, *25*(1), 364.

Hwang, C. Y., Lee, I., Hwang, Y. J., Yoon, S. J., Lee, W. S., & Cho, B. C. (2016). Pseudoalteromonas neustonica sp. nov., isolated from the sea surface microlayer of the Ross Sea (Antarctica), and emended description of the genus Pseudoalteromonas. *International Journal of Systematic and Evolutionary Microbiology*, *66*(9), 3377–3382.

Ivanova, E. P., Gorshkova, N. M., Zhukova, N. V., Lysenko, A. M., Zelepuga, E. A., Prokof’eva, N. G., Mikhailov, V. V., Nicolau, D. V., & Christen, R. (2004). Characterization of Pseudoalteromonas distincta-like sea-water isolates and description of Pseudoalteromonas aliena sp. nov. *International Journal of Systematic and Evolutionary Microbiology*, *54*(Pt 5), 1431–1437.

Ivanova, E. P., Sawabe, T., Lysenko, A. M., Gorshkova, N. M., Hayashi, K., Zhukova, N. V., Nicolau, D. V., Christen, R., & Mikhailov, V. V. (2002). Pseudoalteromonas translucida sp. nov. and Pseudoalteromonas paragorgicola sp. nov., and emended description of the genus. *International Journal of Systematic and Evolutionary Microbiology*, *52*(Pt 5), 1759–1766.

Ivanova, E. P., Sawabe, T., Lysenko, A. M., Gorshkova, N. M., Svetashev, V. I., Nicolau, D. V., Yumoto, N., Taguchi, T., Yoshikawa, S., Christen, R., & Mikhailov, V. V. (2002). Pseudoalteromonas ruthenica sp. nov., isolated from marine invertebrates. *International Journal of Systematic and Evolutionary Microbiology*, *52*(Pt 1), 235–240.

Jo, J., Choi, H., Lee, S.-G., Oh, J., Lee, H.-G., & Park, C. (2017). Draft Genome Sequences of Pseudoalteromonas tetraodonis CSB01KR and Pseudoalteromonas lipolytica CSB02KR, Isolated from the Gut of the Sea Cucumber *Apostichopus japonicus*. *Genome Announcements*, *5*(28). https://doi.org/10.1128/genomea.00627-17

Kitaguchi, H., Masu, N., Fujii, K., & Mitsutani, A. (2019). Draft genome sequence of Pseudoalteromonas sp. Strain A25, a bacterium with algicidal activity against diatoms. *Microbiology Resource Announcements*, *8*(45). https://doi.org/10.1128/MRA.01254-19

Klassen, J. L., Rischer, M., Wolf, T., Guo, H., Shelest, E., Clardy, J., & Beemelmanns, C. (2015). Genome sequences of three Pseudoalteromonas strains (P1-8, P1-11, and P1-30), isolated from the marine hydroid Hydractinia echinata. *Genome Announcements*, *3*(6). https://doi.org/10.1128/genomeA.01380-15

Klassen, J. L., Wolf, T., Rischer, M., Guo, H., Shelest, E., Clardy, J., & Beemelmanns, C. (2015). Draft genome sequences of six Pseudoalteromonas strains, P1-7a, P1-9, P1-13-1a, P1-16-1b, P1-25, and P1-26, which induce larval settlement and metamorphosis in Hydractinia echinata. *Genome Announcements*, *3*(6). https://doi.org/10.1128/genomeA.01477-15

Kudo, R., Yamano, R., Yu, J., Hatakeyama, S., Jiang, C., Mino, S., Yamaki, S., Ando, Y., Sakai, Y., & Sawabe, T. (2024). The Description of Pseudoalteromonas apostichopi sp. nov., Vibrio apostichopi sp. nov., and Marinobacter apostichopi sp. nov. from the Fertilized Eggs and Larvae of Apostichopus japonicus. *Current Microbiology*, *81*(8), 246.

Li, B., Wang, P., Zeng, Z., Cai, X., Wang, G., & Wang, X. (2016). Complete genome sequence of Pseudoalteromonas rubra SCSIO 6842, harboring a putative conjugative plasmid pMBL6842. *Journal of Biotechnology*, *224*, 66–67.

Machado, H., Sonnenschein, E. C., Melchiorsen, J., & Gram, L. (2015). Genome mining reveals unlocked bioactive potential of marine Gram-negative bacteria. *BMC Genomics*, *16*(1), 158.

Mead, O. L., Hahn, E. E., & Adamska, M. A. (2021). Hybrid genome assemblies of marine bacteria isolated from the sponge Sycon capricorn. *Microbiology Resource Announcements*, *10*(43), e0085821.

Meyer JL, J. K. (2024). Development of alternative in situ treatments for stony coral tissue loss disease. *DEP Reports. Florida.*, 13 pp.

Mikkelsen, M. D., Cao, H. T. T., Roret, T., Rhein-Knudsen, N., Holck, J., Tran, V. T. T., Nguyen, T. T., Tran, V. H. N., Lezyk, M. J., Muschiol, J., Pham, T. D., Czjzek, M., & Meyer, A. S. (2021). A novel thermostable prokaryotic fucoidan active sulfatase PsFucS1 with an unusual quaternary hexameric structure. *Scientific Reports*, *11*(1), 19523.

Nam, Y.-D., Chang, H.-W., Park, J. R., Kwon, H.-Y., Quan, Z.-X., Park, Y.-H., Lee, J.-S., Yoon, J.-H., & Bae, J.-W. (2007). Pseudoalteromonas marina sp. nov., a marine bacterium isolated from tidal flats of the Yellow Sea, and reclassification of Pseudoalteromonas sagamiensis as Algicola sagamiensis comb. nov. *International Journal of Systematic and Evolutionary Microbiology*, *57*(Pt 1), 12–18.

Nedashkovkaya, O. I., Kim, S.-G., Balabanova, L. A., Zhukova, N. V., Son, O. M., Tekutyeva, L. A., & Mikhailov, V. V. (2021). Genome-based classification of strain 16-SW-7, a marine bacterium capable of converting B red blood cells, as Pseudoalteromonas distincta and proposal to reclassify Pseudoalteromonas paragorgicola as a later heterotypic synonym of Pseudoalteromonas distincta. *Frontiers in Microbiology*, *12*, 809431.

Paulsen, S. S., Isbrandt, T., Kirkegaard, M., Buijs, Y., Strube, M. L., Sonnenschein, E. C., Larsen, T. O., & Gram, L. (2020). Production of the antimicrobial compound tetrabromopyrrole and the Pseudomonas quinolone system precursor, 2-heptyl-4-quinolone, by a novel marine species Pseudoalteromonas galatheae sp. nov. *Scientific Reports*, *10*(1), 21630.

Paulsen, S. S., Strube, M. L., Bech, P. K., Gram, L., & Sonnenschein, E. C. (2019). Marine chitinolytic Pseudoalteromonas represents an untapped reservoir of bioactive potential. *MSystems*, *4*(4). https://doi.org/10.1128/mSystems.00060-19

Phillips, E. K., Shaffer, J. M. C., Henson, M. W., Coelho, J. T., Martin, M. O., & Thrash, J. C. (2023). Genome sequences of four agarolytic bacteria from the Bacteroidia and Gammaproteobacteria. *Microbiology Resource Announcements*, *12*(11), e0066723.

Rajeev, M., Sushmitha, T. J., Toleti, S. R., & Pandian, S. K. (2021). Draft genome sequencing of Pseudoalteromonas tetraodonis strain kknpp56, a potent biofilm-forming bacterium isolated from early-stage marine biofilm. *Microbiology Resource Announcements*, *10*(38). https://doi.org/10.1128/mra.00605-21

Romanenko, L. A., Zhukova, N. V., Rohde, M., Lysenko, A. M., Mikhailov, V. V., & Stackebrandt, E. (2003). Pseudoalteromonas agarivorans sp. nov., a novel marine agarolytic bacterium. *International Journal of Systematic and Evolutionary Microbiology*, *53*(Pt 1), 125–131.

Rong, J.-C., Liu, M., Li, Y., Sun, T.-Y., Pang, X.-H., Qin, Q.-L., Chen, X.-L., & Xie, B.-B. (2016). Complete genome sequence of a marine bacterium with two chromosomes, Pseudoalteromonas translucida KMM 520T. *Marine Genomics*, *26*, 17–20.

Rosado, P. M., Cardoso, P. M., Rosado, J. G., Schultz, J., Nunes da Rocha, U., Keller-Costa, T., & Peixoto, R. S. (2023). Exploring the potential molecular mechanisms of interactions between a probiotic consortium and its coral host. *MSystems*, *8*(1). https://doi.org/10.1128/msystems.00921-22

Rosario, M. E., Camm, J., Cavanagh, D., Rowley, D. C., & Nelson, D. R. (2021). Draft Genome Sequence of Pseudoalteromonas sp. Strain JC3. *Microbiology Resource Announcements*, *10*(36), e0021221.

Sakai-Kawada, F. E., Ip, C. G., Hagiwara, K. A., Nguyen, H.-Y. X., Yakym, C.-J. A. V., Helmkampf, M., Armstrong, E. E., & Awaya, J. D. (2020). Characterization of Prodiginine Pathway in Marine Sponge-Associated Pseudoalteromonas sp. PPB1 in Hilo, Hawai‘i. *Frontiers in Sustainable Food Systems*, *4*. https://doi.org/10.3389/fsufs.2020.600201

Suria, A. M., Tan, K. C., Kerwin, A. H., Gitzel, L., Abini-Agbomson, L., Bertenshaw, J. M., Sewell, J., Nyholm, S. V., & Balunas, M. J. (2020). Hawaiian bobtail squid symbionts inhibit marine bacteria via production of specialized metabolites, including new bromoalterochromides BAC-D/D’. *MSphere*, *5*(4). https://doi.org/10.1128/mSphere.00166-20

Sweet, M., Villela, H., Keller-Costa, T., Costa, R., Romano, S., Bourne, D. G., Cárdenas, A., Huggett, M. J., Kerwin, A. H., Kuek, F., Medina, M., Meyer, J. L., Müller, M., Pollock, F. J., Rappé, M. S., Sere, M., Sharp, K. H., Voolstra, C. R., Zaccardi, N., … Peixoto, R. (2021). Insights into the cultured bacterial fraction of corals. *MSystems*, *6*(3), e0124920.

Taguchi, M., Guo, Y., Nishizawa, T., Chohnan, S., & Kurusu, Y. (2022). Complete genome sequence of a psychrophilic bacterium, Pseudoalteromonas sp. Strain APM04, isolated from the seafloor of the South Mariana Trough, Pacific Ocean. *Microbiology Resource Announcements*, *11*(8), e0037422.

Tang, B.-L., Rong, J.-C., Dang, Y.-R., Xie, B.-B., Chen, X.-L., & Zhang, X.-Y. (2018). Complete genomic sequence of Pseudoalteromonas sp. Strain SAO4-4, a protease-producing bacterium isolated from seawater of the Atlantic ocean. *Genome Announcements*, *6*(22). https://doi.org/10.1128/genomeA.00284-18

Tang, K., Zhan, W., Zhou, Y., Xu, T., Chen, X., Wang, W., Zeng, Z., Wang, Y., & Wang, X. (2020). Antagonism between coral pathogen Vibrio coralliilyticus and other bacteria in the gastric cavity of scleractinian coral Galaxea fascicularis. *Science China Earth Sciences*, *63*(1), 157–166.

Thomas, E. M., Smith, E. M., Papke, E., Shlafstein, M. D., Grant-Beurmann, S., Coelho, L. C., Trott, A. C., Häse, C. C., Oline, D. K., Videau, P., Saw, J. H., Strangman, W. K., & Ushijima, B. (2025). Pseudoalteromonas ardens sp. nov., Pseudoalteromonas obscura sp. nov. and Pseudoalteromonas umbrosa sp. nov. isolated from the coral Montipora capitata on a reef in Kāne’ohe Bay, O’ahu, Hawai’i. *International Journal of Systematic and Evolutionary Microbiology*, *75*(2). https://doi.org/10.1099/ijsem.0.006681

Thomas, T., Evans, F. F., Schleheck, D., Mai-Prochnow, A., Burke, C., Penesyan, A., Dalisay, D. S., Stelzer-Braid, S., Saunders, N., Johnson, J., Ferriera, S., Kjelleberg, S., & Egan, S. (2008). Analysis of the Pseudoalteromonas tunicata genome reveals properties of a surface-associated life style in the marine environment. *PloS One*, *3*(9), e3252.

Ushijima, B., Gunasekera, S. P., Meyer, J. L., Tittl, J., Pitts, K. A., Thompson, S., Sneed, J. M., Ding, Y., Chen, M., Jay Houk, L., Aeby, G. S., Häse, C. C., & Paul, V. J. (2023). Chemical and genomic characterization of a potential probiotic treatment for stony coral tissue loss disease. *Communications Biology*, *6*(1), 248.

Varasteh, T., Hamerski, L., Tschoeke, D., Lima, A. S., Garcia, G., Cosenza, C. A. N., Thompson, C., & Thompson, F. (2021). Conserved Pigment Profiles in Phylogenetically Diverse Symbiotic Bacteria Associated with the Corals Montastraea cavernosa and Mussismilia braziliensis. *Microbial Ecology*, *81*(1), 267–277.

Videau, P., Shlafstein, M. D., Oline, D. K., Givan, S. A., Chapman, L. F., Strangman, W. K., Hahnke, R. L., Saw, J. H., & Ushijima, B. (2024). Genome-based taxonomic analysis of the genus Pseudoalteromonas reveals heterotypic synonyms. *Environmental Microbiology*, *26*(7), e16672.

Villela, H., Modolon, F., Schultz, J., Delgadillo-Ordoñez, N., Carvalho, S., Soriano, A. U., & Peixoto, R. S. (2023). Genome analysis of a coral-associated bacterial consortium highlights complementary hydrocarbon degradation ability and other beneficial mechanisms for the host. *Scientific Reports*, *13*(1). https://doi.org/10.1038/s41598-023-38512-z

Wang, P., Zeng, Z., Wang, W., Wen, Z., Li, J., & Wang, X. (2017). Dissemination and loss of a biofilm-related genomic island in marine Pseudoalteromonas mediated by integrative and conjugative elements. *Environmental Microbiology*, *19*(11), 4620–4637.

Wang, W., Tang, K., Wang, P., Zeng, Z., Xu, T., Zhan, W., Liu, T., Wang, Y., & Wang, X. (2022). The coral pathogen Vibrio coralliilyticus kills non-pathogenic holobiont competitors by triggering prophage induction. *Nature Ecology & Evolution*, *6*(8), 1132–1144.

Worden, P. J., Bogema, D. R., Micallef, M. L., Go, J., Deutscher, A. T., Labbate, M., Green, T. J., King, W. L., Liu, M., Seymour, J. R., & Jenkins, C. (2022). Phylogenomic diversity of Vibrio species and other Gammaproteobacteria isolated from Pacific oysters (Crassostrea gigas) during a summer mortality outbreak. *Microbial Genomics*, *8*(12). https://doi.org/10.1099/mgen.0.000883

Wu, Y.-H., Cheng, H., Xu, L., Jin, X.-B., Wang, C.-S., & Xu, X.-W. (2017). Physiological and genomic features of a novel violacein-producing bacterium isolated from surface seawater. *PloS One*, *12*(6), e0179997.

Xie, B-B, Shu, Y.-L., Qin, Q.-L., Rong, J.-C., Zhang, X.-Y., Chen, X.-L., Shi, M., He, H.-L., Zhou, B.-C., & Zhang, Y.-Z. (2012). Genome sequences of type strains of seven species of the marine bacterium Pseudoalteromonas. *Journal of Bacteriology*, *194*(10), 2746–2747.

Xie, Bin-Bin, Shu, Y.-L., Qin, Q.-L., Rong, J.-C., Zhang, X.-Y., Chen, X.-L., Zhou, B.-C., & Zhang, Y.-Z. (2012). Genome sequence of the cycloprodigiosin-producing bacterial strain Pseudoalteromonas rubra ATCC 29570(T). *Journal of Bacteriology*, *194*(6), 1637–1638.

Yan, J., Wu, Y.-H., Meng, F.-X., Wang, C.-S., Xiong, S.-L., Zhang, X.-Y., Zhang, Y.-Z., Xu, X.-W., & Zhang, D.-M. (2016). Pseudoalteromonas gelatinilytica sp. nov., isolated from surface seawater. *International Journal of Systematic and Evolutionary Microbiology*, *66*(9), 3538–3545.

Yang, X., Garuglieri, E., Van Goethem, M. W., Marasco, R., Fusi, M., & Daffonchio, D. (2024). Mangrovimonas cancribranchiae sp. nov., a novel bacterial species associated with the gills of the fiddler crab Cranuca inversa (Brachyura, Ocypodidae) from Red Sea mangroves. *International Journal of Systematic and Evolutionary Microbiology*, *74*(6). https://doi.org/10.1099/ijsem.0.006415

Zeng, Z., Dai, S., Xie, Y., Tian, X., Li, J., & Wang, X. (2014). Genome sequences of two pseudoalteromonas strains isolated from the South china sea. *Genome Announcements*, *2*(2). https://doi.org/10.1128/genomeA.00305-14
