# Supplementary figures and images for: Pseudoalteromonas is a symbiont of marine invertebrates that exhibits broad patterns of phylosymbiosis
Source: ISME J. 2026 Apr 20;20(1):wrag091. doi: 10.1093/ismejo/wrag091 (PMC13245728; doi:10.1093/ismejo/wrag091)

Reference

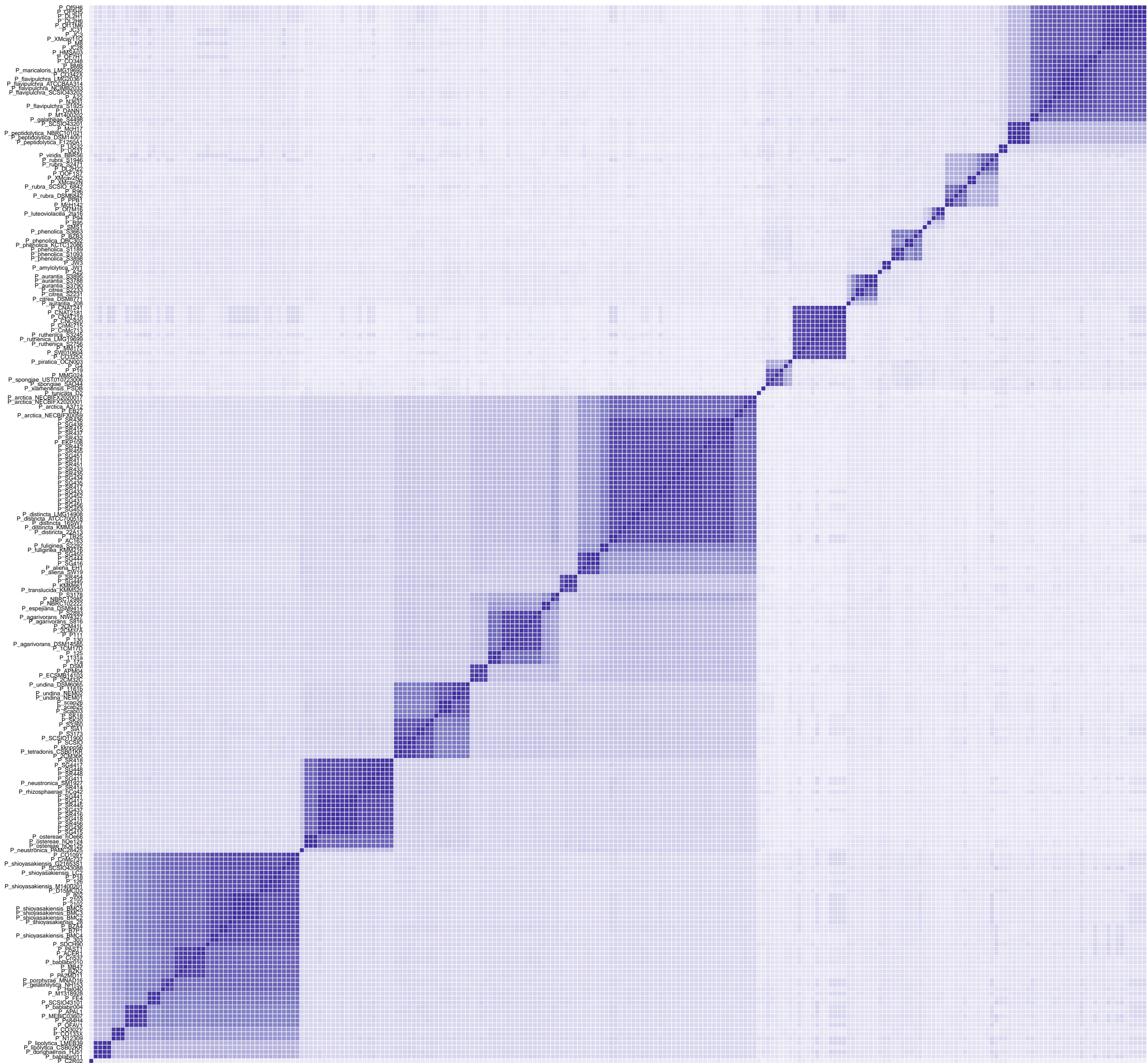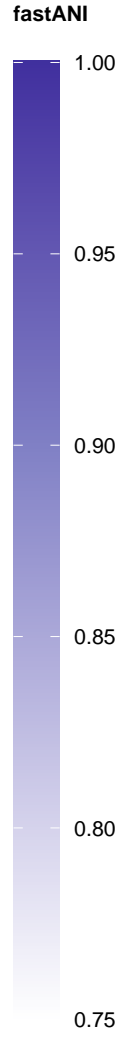

Query

Supplement: Supplementary_material_wrag091 [file supplementary_material_wrag091.zip › 2026-01-14-extended-figure-1-review-edits-2.pdf]

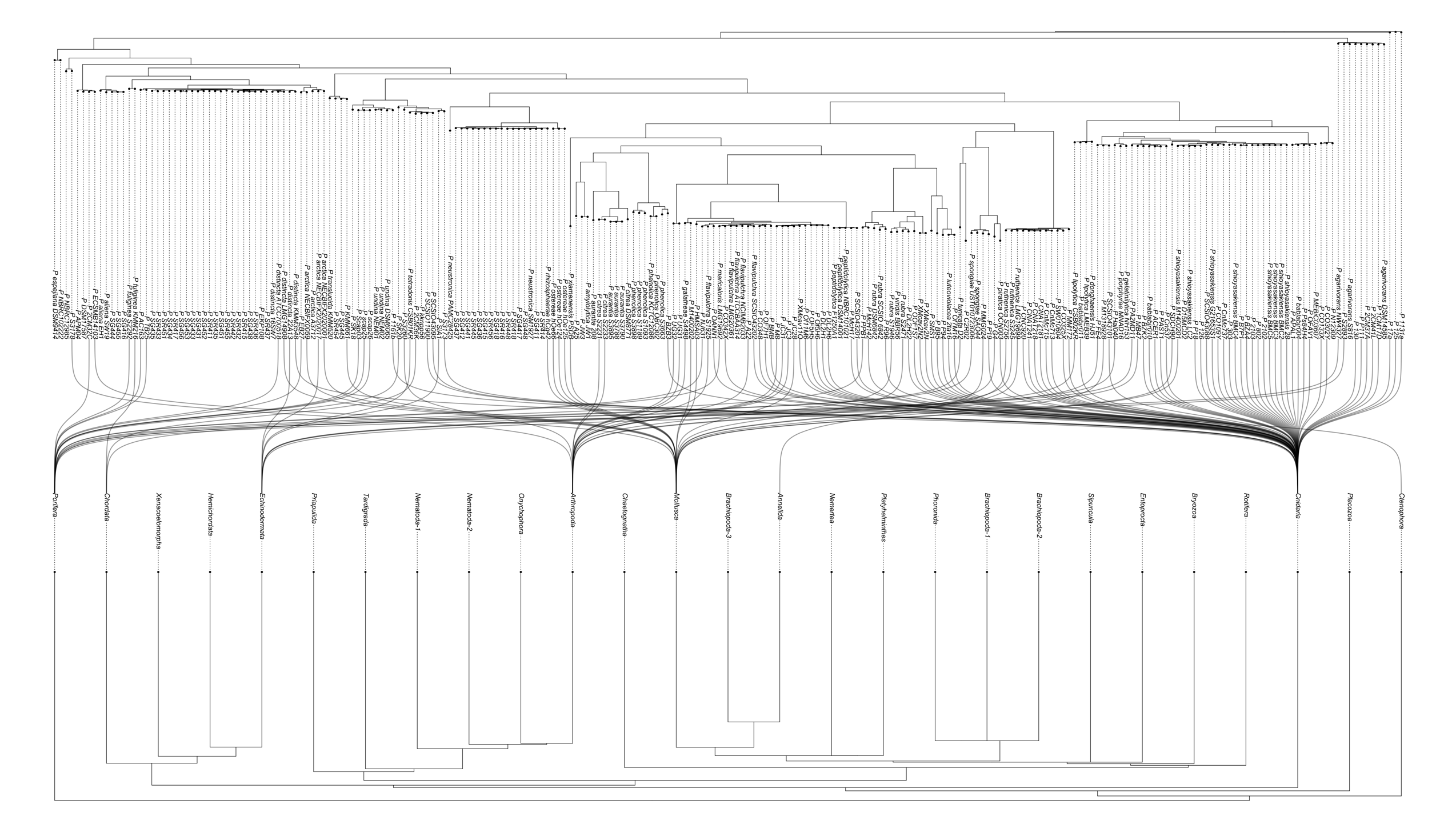

Supplement: Supplementary_material_wrag091 [file supplementary_material_wrag091.zip › 2026-01-14-extended-figure-2-review-edits.png]

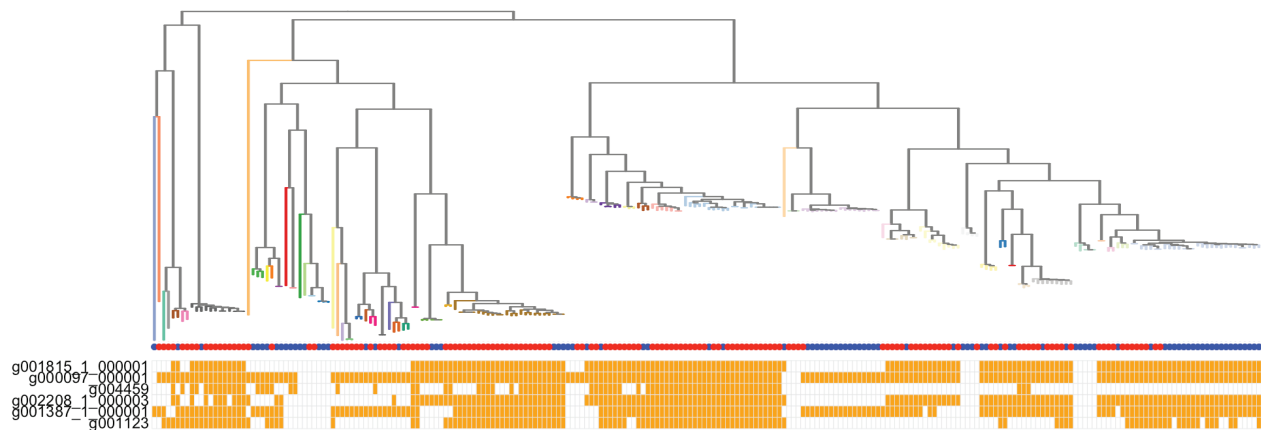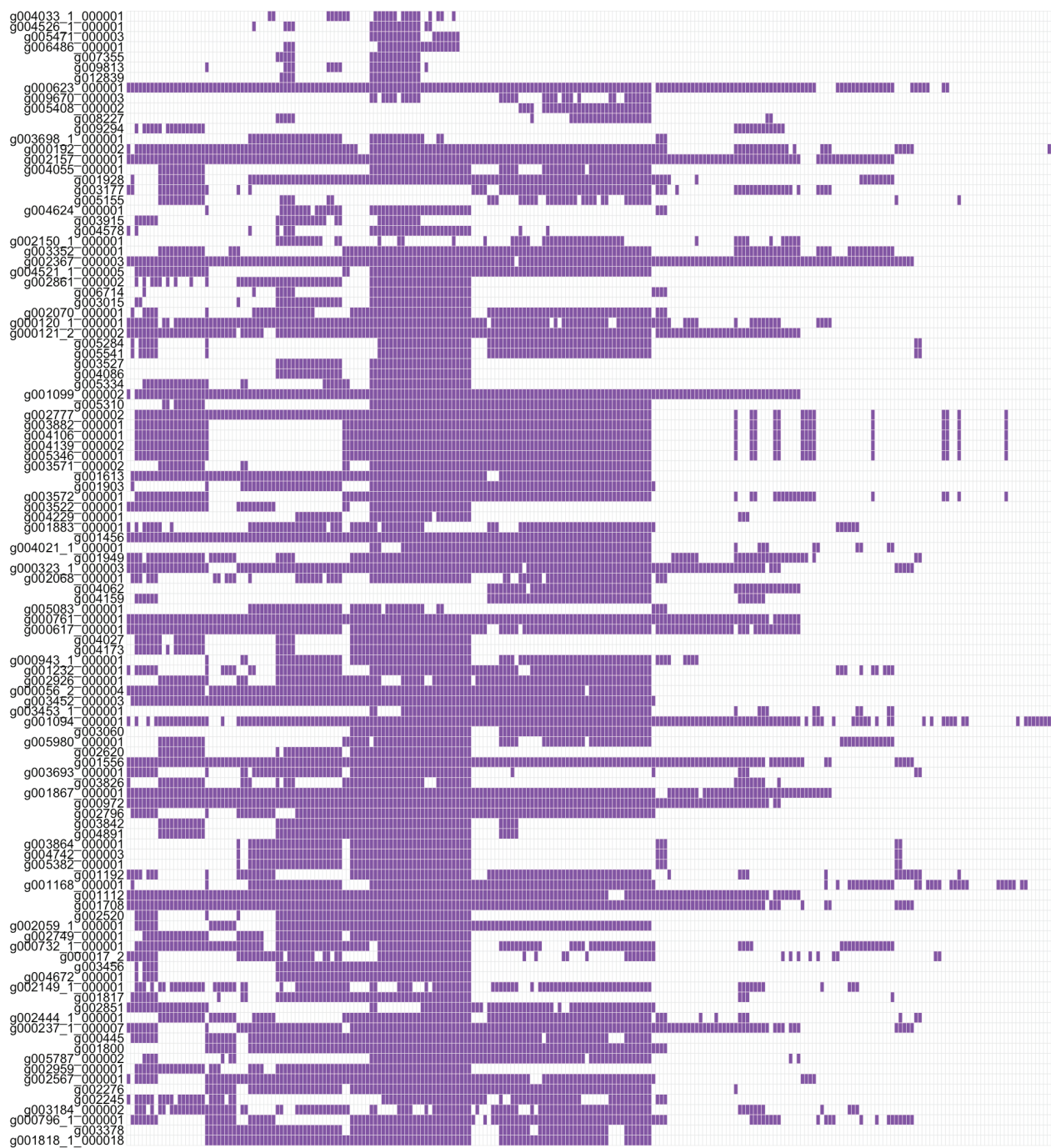

Supplement: Supplementary_material_wrag091 [file supplementary_material_wrag091.zip › 2026-01-14-extended-figure-3-review-edits.pdf]

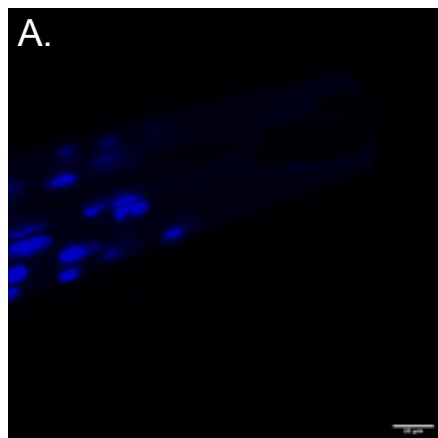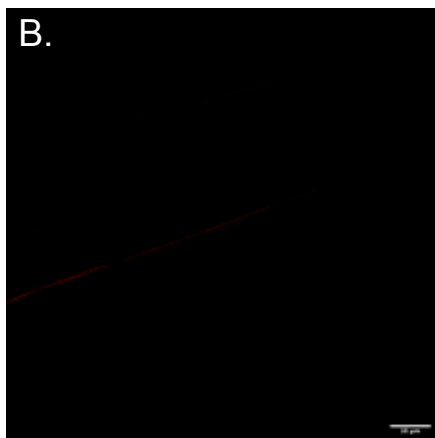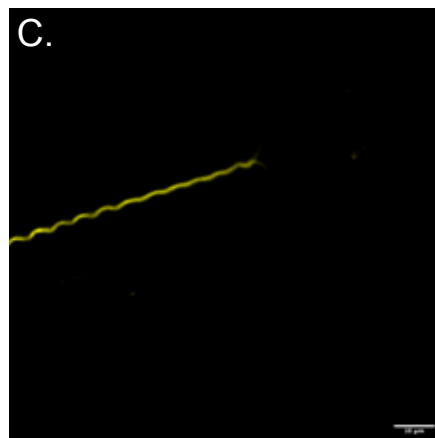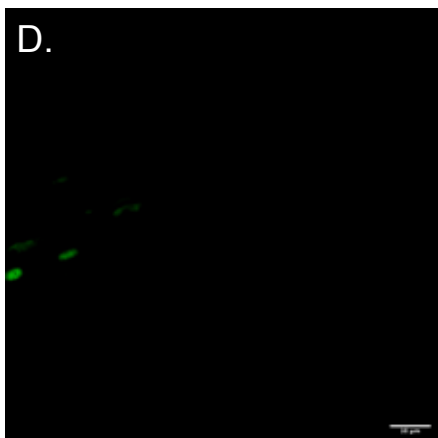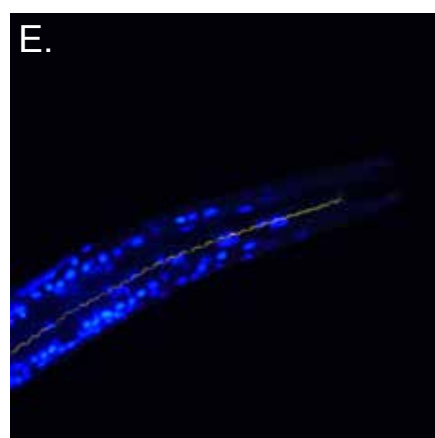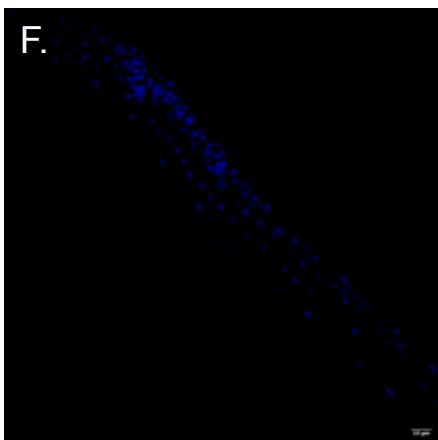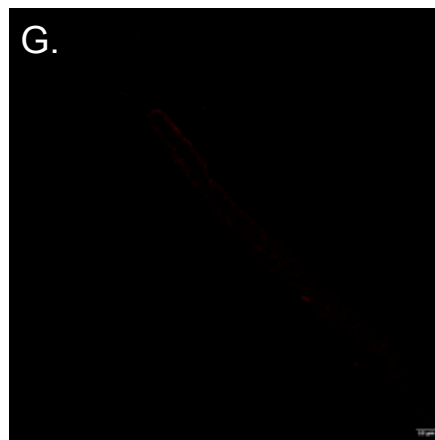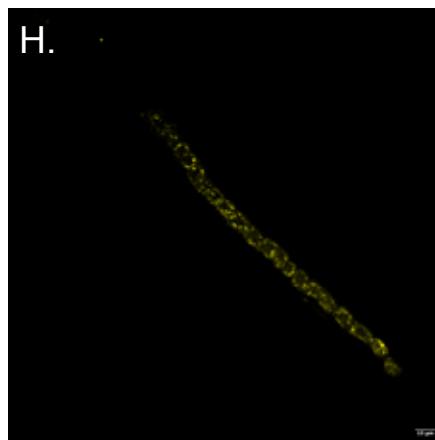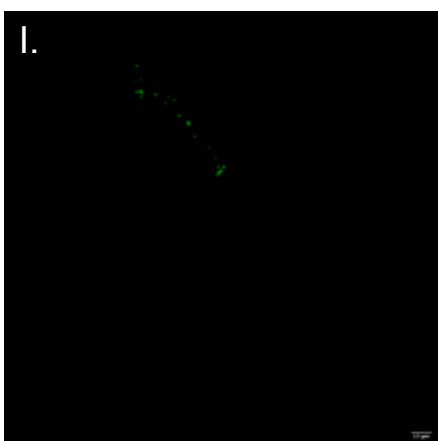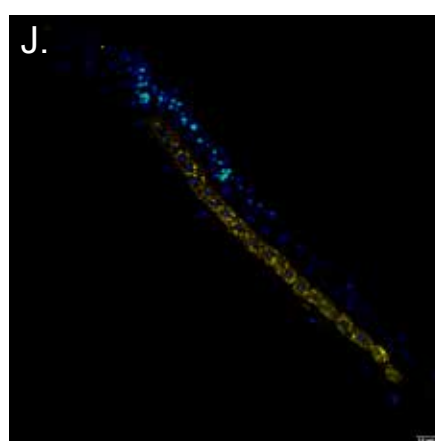

Supplement: Supplementary_material_wrag091 [file supplementary_material_wrag091.zip › 2026-01-14-extended-figure-4-review-edits.pdf]
